# Valorization of icariin *via* α-l-rhamnosidase mutant immobilized on UiO-66-NH_2_: an efficient and recyclable biocatalytic system for icariside I production

**DOI:** 10.1039/d6ra06339j

**Published:** 2026-10-12

**Authors:** Haoyu Jia, Tong Yan, Luran Wang, Lingling Song

**Affiliations:** a School of Pharmacy, Henan University of Chinese Medicine Zhengzhou 450046 China jiahaoyu@hactcm.edu.cn

## Abstract

α-l-Rhamnosidase is a key biocatalyst for glycosidic bond hydrolysis. Icariside I is a high-value flavonoid glycoside with enhanced bioavailability. However, its production from icariin is limited by enzyme stability and suboptimal catalytic performance. Here, an α-l-rhamnosidase mutant (R783A) was heterologously expressed, purified, and immobilized onto UiO-66-NH_2_*via* a simple adsorption strategy to construct a robust biocatalyst. The resulting R783A@UiO-66-NH_2_ exhibited markedly improved stability, including broad pH tolerance (6.0–9.0), retained more than 90% of its initial activity after 2 h at 60 °C, enhanced storage stability (88.6% residual activity after 30 days), and improved resistance to metal ions and organic solvents. Notably, R783A@UiO-66-NH_2_ efficiently catalyzed the hydrolysis of icariin, achieving a 99.8% conversion and a 97.6% icariside I yield within 5 h, corresponding to a space-time yield of 0.52 g L^−1^ h^−1^. In addition, the immobilized biocatalyst retained 85.6% yield after five consecutive reuse cycles and 60.3% after eight cycles at 60 °C, demonstrating useful operational stability and recyclability. This study provides a simple and efficient approach for the biotransformation of icariside I, with UiO-66-NH_2_ nanoparticles offering a versatile platform for high-value flavonoid production.

## Abstract 2

Immobilized α-l-rhamnosidase mutant R783A catalyzes glycosidic bond hydrolysis with excellent reusability.

## Graphical abstract

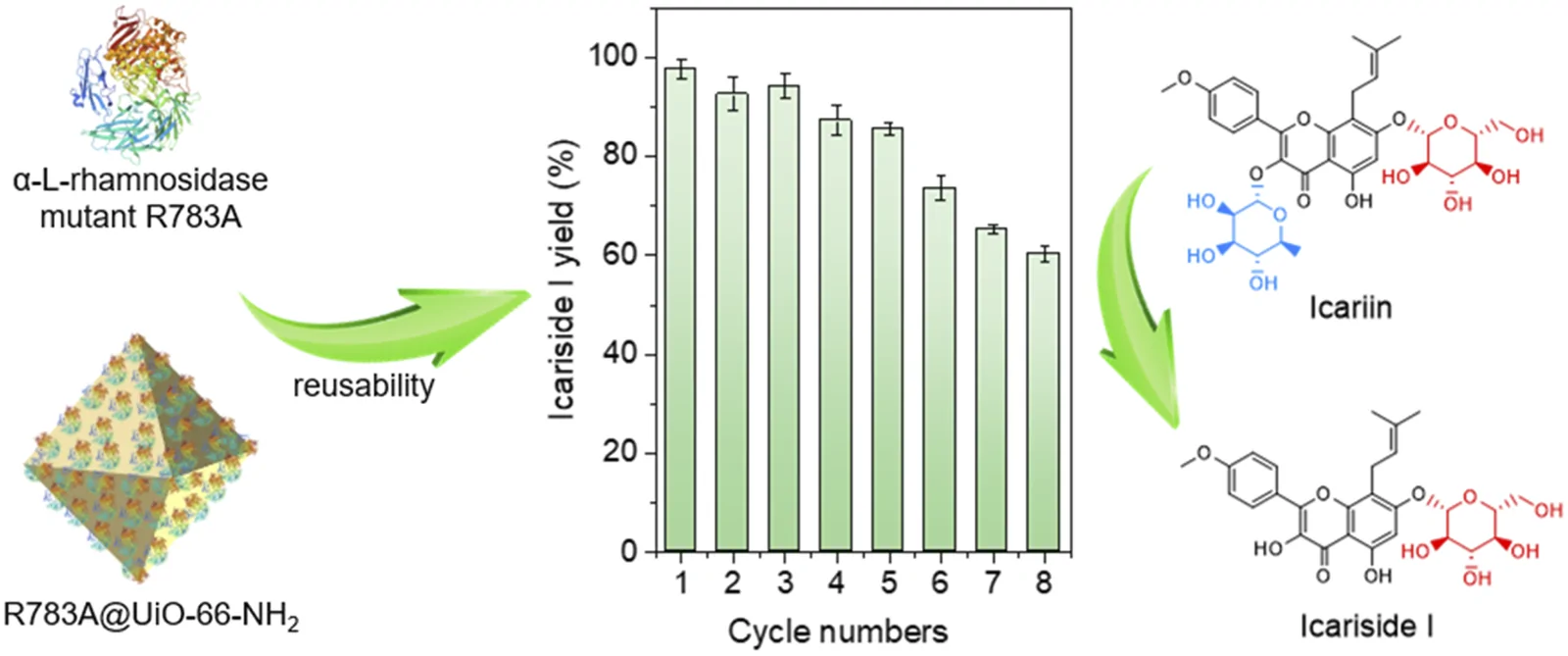

## 1. Introduction

Icariside I, a pharmacologically active flavonoid glycoside from traditional Chinese medicinal herb *Herba Epimedii*,^1^ has attracted considerable attention in the pharmaceutical field owing to its enhanced bioavailability and diverse therapeutic properties, including anti-osteoporotic, neuroprotective, and anticancer activities.^2–6^ Traditionally, icariside I was obtained either by direct extraction from *Epimedium* species or through chemical hydrolysis of icariin. However, the natural abundance of icariside I in plants is extremely low, making direct extraction inefficient, time-consuming, and economically unfavorable for large-scale production.^7^ Meanwhile, conventional chemical hydrolysis methods often require strong acids, high temperatures, or prolonged reaction times, which may result in poor regioselectivity, undesired side reactions, low product purity, and environmental pollution.^8^ These drawbacks greatly limit the practical application and industrial scalability of traditional preparation methods. In recent years, enzymatic transformation strategies have emerged as promising alternatives due to their high specificity, mild conditions, and environmental benignity.^9–11^ Compared with chemical methods, they enable selective glycosidic bond hydrolysis with fewer by-products and lower energy consumption. Therefore, developing an efficient and green enzymatic system for converting icariin to icariside I is of significant scientific and industrial importance.

α-l-Rhamnosidase (EC 3.2.1.40) is a glycoside hydrolase widely distributed in bacteria and fungi that catalyzes the cleavage of terminal α-l-rhamnose residues from various natural glycosides, particularly flavonoid glycosides.^12^ Due to its high regioselectivity in modifying glycosylation patterns, this enzyme has attracted considerable interest for applications in the food and pharmaceutical industries.^13,14^ However, the industrial application of such biocatalysts remains limited by several inherent drawbacks. In particular, the free enzymes generally exhibit poor operational stability and are highly sensitive to environmental conditions such as temperature and pH. In addition, their difficulty in recovery and reuse significantly increases production costs,^15,16^ which ultimately restricts their large-scale application in flavonoid biotransformation processes.

To address these limitations, enzyme immobilization has emerged as an effective strategy to enhance biocatalyst stability and reusability. Among various immobilization supports, metal–organic frameworks (MOFs) are particularly attractive due to their high porosity, structural tunability, and large surface area.^17,18^ Recent studies have demonstrated the significant potential of MOF-based biocomposites in flavonoid and glycoside biotransformation. For instance, a β-glucosidase@MOF biocomposite was constructed through a one-pot coprecipitation strategy, achieving 99.8% conversion of icariin to baohuoside I while retaining 70.5% residual activity after eight reuse cycles.^19^ Similarly, Wang *et al.* immobilized α-l-rhamnosidase on magnetic MOFs, resulting in 91.42% conversion of rutin to isoquercitrin, enhanced substrate affinity, and 73.55% residual activity after 30 cycles at 60 °C.^20^ Sun *et al.* further developed a cellulase@UiO-66-NH_2_ biocomposite *via* mechanochemical biomimetic mineralization, which exhibited improved catalytic efficiency and maintained over 50% activity after eight cycles.^21^ In addition, Li *et al.* immobilized glucosyltransferases on mesoporous Zn–Ni MOFs through metal chelation, enabling one-step enzyme purification and efficient catalytic conversion of ginsenoside Rh2, while preserving 76% residual activity after seven cycles and 80.22% activity after 45 days of storage.^22^ Therefore, MOF-based enzyme immobilization represents an effective and promising strategy for improving the stability, robustness, and reusability of biocatalysts for the high-value production of bioactive natural products.

In this study, the α-l-rhamnosidase mutant R783A derived from *Dictyoglomus thermophilum* was successfully expressed, purified, and immobilized onto Zr-based MOF (UiO-66-NH_2_) through physical adsorption. The immobilization conditions were optimized to maximize enzyme loading and catalytic performance. The resulting biocomposite, R783A@UiO-66-NH_2_, was characterized to evaluate its structural features and catalytic properties. We systematically evaluated the enzymatic properties of R783A@UiO-66-NH_2_ in comparison to its free counterpart, focusing on critical parameters such as pH stability, thermal tolerance, kinetic behavior, and operational stability. The immobilized enzyme exhibited significantly enhanced stability and favorable catalytic characteristics. Furthermore, the practical applicability of R783A@UiO-66-NH_2_ was demonstrated through the efficient biotransformation of icariin to icariside I, where it displayed useful recyclability. These findings enrich the current understanding of MOF-based enzyme immobilization and offer a reusable biocatalytic system for the high-value modification of flavonoids.

## 2. Materials and methods

### 2.1. Materials and chemicals

*p*-Nitrophenol (*p*NP), *p*-nitrophenyl-α-l-rhamnopyranoside (*p*NPR), icariin, icariside I, IPTG, and kanamycin were purchased from Macklin (Shanghai, China). The chemicals for preparing MOFs including 2-aminobenzenedicarboxylic acid, *N*,*N*-dimethylformamide (DMF) and zirconium chloride (ZrCl_4_) were obtained from Aladdin Chemicals (Shanghai, China). Copper Assay Kit (Colorimetric) and Zinc Assay Kit (Colorimetric) were purchased from Beyotime (Shanghai, China). All chemicals were purchased from commercial sources and directly used.

### 2.2. Preparation of α-l-rhamnosidase R783A and activity assay

The codon-optimized gene sequence of wild-type α-l-rhamnosidase from *Dictyoglomus thermophilum* (GenBank: WP_012548615) was synthesized by Genscript (Nanjing, China). Site-directed mutagenesis was performed to generate the R783A mutant, which was then heterologously expressed using the recombinant plasmid pET-28a in *E. coli* BL21(DE3). The recombinant plasmid construction and enzyme purification procedures were performed according to a previously reported method.^23^ Cells were cultured in LB medium with kanamycin (50 µg mL^−1^) at 37 °C until OD600 reached 0.6–0.8, then induced with 0.1 mM IPTG and incubated at 20 °C for 20 h. Cells were harvested, resuspended in binding buffer (20 mM sodium phosphate, 500 mM NaCl, 10 mM imidazole; pH 7.4), and lysed by ultrasonication (30% amplitude, 2 s on/3 s off, 15 min, 4 °C). After centrifugation, the supernatant was applied to a HisTrap™ HP column and eluted with buffer containing 500 mM imidazole. The eluted protein was desalted using a desalting column and analyzed by SDS-PAGE.

Enzyme activity was determined by monitoring the initial linear increase in absorbance at 405 nm during the first 3 min using a UV-vis spectrophotometer. One unit (U) of enzyme activity was defined as the amount of enzyme required to catalyze the formation of 1 µmol of *p*-nitrophenol per minute under the assay conditions. Protein concentrations were determined using the Bradford assay^24^ with bovine serum albumin (BSA) as the standard.

### 2.3. Synthesis of the UiO-66-NH_2_

The synthesis of UiO-66-NH_2_ was carried out following a reported procedure with minor modifications.^25^ ZrCl_4_ (1.491 g) and 2-aminoterephthalic acid (1.146 g) were dissolved in 100 mL of DMF to obtain a homogeneous solution, which was then equally divided into two 100 mL Teflon-lined autoclaves. The sealed autoclaves were heated at 120 °C for 24 h and subsequently cooled to room temperature. The resulting precipitate was collected, washed three times with ethanol, and dried at 60 °C for further use.

### 2.4. Synthesis of R783A@UiO-66-NH_2_

R783A@UiO-66-NH_2_ was prepared *via* a physical adsorption method.^26^ Briefly, 20 mg UiO-66-NH_2_ was dispersed in 1.5 mL of deionized water, followed by the addition of 0.5 mL of R783A solution (1 mg mL^−1^). The suspension was stirred at room temperature for 3 h and then incubated at 4 °C for 1 h. The resulting precipitate was collected by centrifugation (4835×*g*, 4 min), washed three times with deionized water, and stored at 4 °C for further characterization.

### 2.5. Optimization of immobilization parameters

To enhance the immobilization efficiency of R783A@UiO-66-NH_2_, the effects of UiO-66-NH_2_ dosage and immobilization time on enzyme loading and activity recovery were systematically investigated. For optimization of UiO-66-NH_2_ dosage, different amounts of UiO-66-NH_2_ (5, 10, 15, 20, 25, 30, and 35 mg) were incubated with 0.5 mg of R783A in 2 mL of Tris–HCl buffer (100 mM, pH 8.0). The mixtures were stirred at 25 °C for 3 h, followed by incubation at 4 °C for 1 h. For optimization of immobilization time, 20 mg of UiO-66-NH_2_ was mixed with 2 mL of R783A solution (0.25 mg mL^−1^) and incubated at 25 °C for varying durations (0.5–6 h). After immobilization, the suspensions were centrifuged to separate the supernatant from the precipitate. The concentration of unbound enzyme in the supernatant was determined using the Bradford assay. The enzyme loading calculated using eqn (1) refers to the amount of enzyme protein actually immobilized per unit mass of the support material. It represents the carrying capacity of the carrier under specific conditions and is typically expressed in mg of enzyme per g of support (mg g^−1^). The immobilization yield calculated using eqn (2) is defined as the percentage of the initially offered enzyme that is successfully immobilized onto the support material. The recovery activity, defined as the ratio of the total activity of R783A@UiO-66-NH_2_ to the total activity of free R783A used to prepare R783A@UiO-66-NH_2_, was determined using eqn (3).

1 Enzyme loading (mg g^−1^) = (*M*_0_ − *M*_1_)/*M*

2 Immobilization yield (%) = (*M*_0_ − *M*_1_)/*M*_0_ × 100

3 Recovery activity (%) = *A*_1_/*A*_0_ × 100

where the *M*_0_ is the initial mass of enzyme offered for immobilization, *M*_1_ is the mass of unbound enzyme in the supernatant after immobilization, *M* is the mass of UiO-66-NH_2_ material, *A*_0_ is the total activity of the free enzyme initially offered and *A*_1_ is the total activity of the immobilized enzyme, both activities are normalized to the same amount of protein offered.

### 2.6. Characterization methods

The morphological characteristics of UiO-66-NH_2_ and R783A@UiO-66-NH_2_ were characterized by scanning electron microscopy (SEM) (Sigma 300, ZEISS, Germany), and their chemical composition was analyzed using an energy-dispersive spectrometer (EDS) (Xplore30, Oxford Instruments, UK). The crystal structures were analyzed by X-ray diffraction (XRD) using a Bruker D8 ADVANCE diffractometer (Germany) with Cu Kα radiation (*λ* = 1.5418 Å), operated at 40 kV and 40 mA over a 2*θ* range of 4–80°. Fourier-transform infrared (FTIR) spectra were recorded on a Bruker INVENIO spectrometer with KBr pellets in the range of 500–4000 cm^−1^. Prior to sample measurement, the background spectrum of blank UiO-66-NH_2_ was collected under identical instrumental conditions and subtracted from the spectrum of R783A@UiO-66-NH_2_ to eliminate interference from intrinsic vibrational bands of the support. Thermal stability was evaluated by thermogravimetric analysis (TGA) using a TGA550 thermogravimetric analyzer (TA Instruments, USA), with samples heated from 20 to 600 °C under a nitrogen atmosphere at a heating rate of 10 °C min^−1^.

### 2.7. Enzymatic properties

#### 2.7.1. Optimization of temperature and pH

The enzymatic properties of free R783A and R783A@UiO-66-NH_2_ were characterized using *p*NPR as the substrate under standard assay conditions at equal protein mass. The optimal reaction temperature was assessed by measuring enzyme activity at temperatures ranging from 40 to 90 °C in Tris–HCl buffer (100 mM, pH 8.0). The optimal pH was determined at 60 °C over a pH range of 3.0–9.0 using appropriate buffer systems, including citrate–phosphate buffer (100 mM, pH 3.0–6.0), PB buffer (100 mM, pH 6.0–8.0) and Tris–HCl buffer (100 mM, pH 8.0–9.0). All assays were performed in triplicate.

#### 2.7.2. pH, thermal and storage stability

The pH and thermal stability of free R783A and R783A@UiO-66-NH_2_ were assessed by measuring residual activity following pre-incubation under defined conditions. For pH stability, the enzymes were incubated in buffers ranging from pH 3.0 to 9.0 at 25 °C for 2 h. Thermal stability was evaluated by incubating the enzymes in Tris–HCl buffer (100 mM, pH 8.0) at temperatures from 60 °C for 2 h. After pre-incubation, residual activity was determined using the standard assay and expressed as a percentage of the initial activity measured immediately before pre-incubation, which was defined as 100%.

Storage stability was further investigated by maintaining both free and immobilized R783A in Tris–HCl buffer (pH 8.0, 100 mM) at 4 °C for 30 days. Enzyme activity was measured at regular intervals to monitor stability over time, with initial activities of both forms normalized to 100% for comparison.

#### 2.7.3. Effects of metal ions and organic reagents on enzyme activity

The activity of free R783A and R783A@UiO-66-NH_2_ to metal ions and organic solvents was evaluated by measuring residual enzyme activity under various conditions. The effects of metal ions and other reagents on free R783A and R783A@UiO-66-NH_2_ activity were assessed in the presence of 1 mM of Na^+^, K^+^, Ca^2+^, Cu^2+^, Mg^2+^, Zn^2+^, Mn^2+^, Fe^2+^, Fe^3+^, dithiothreitol (DTT), and ethylenediaminetetraacetic acid (EDTA). To examine effect of organic solvent, enzyme activity was measured in the presence of methanol, ethanol, dimethyl sulfoxide (DMSO), and acetonitrile (ACN) at final concentrations of 10% (v/v).

The adsorption of metal ions by R783A@UiO-66-NH_2_ was evaluated using colorimetric assay kits for Cu^2+^ and Zn^2+^. R783A@UiO-66-NH_2_ (0.5 U mL^−1^) was incubated with 1 mM Cu^2+^ or Zn^2+^ solution in Tris–HCl buffer (100 mM, pH 8.0) for a designated period. After incubation, the mixture was centrifuged to remove the biocatalyst. The concentrations of Cu^2+^ and Zn^2+^ in the diluted supernatant were then determined using the corresponding colorimetric assay kits according to the manufacturer's instructions. The metal-ion concentration in a control sample without R783A@UiO-66-NH_2_ was measured as control. All measurements were performed in triplicate.

#### 2.7.4. Catalytic kinetic parameters

To determine the kinetic parameters of free R783A and R783A@UiO-66-NH_2_ toward *p*NPR, a series of substrate concentrations (0.1–5.0 mM) was employed. Initial reaction rates were measured under optimal reaction conditions for both free and immobilized enzymes. The kinetic parameters, including *K*_m_, *V*_max_, *k*_cat_, and *k*_cat_/*K*_m_, were calculated based on the Michaelis–Menten equation:4
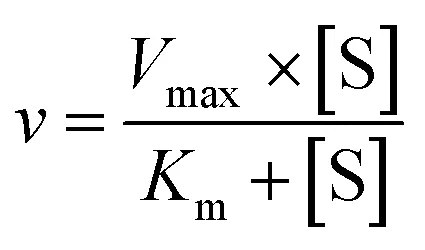
5
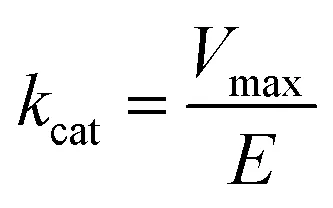
where [S] is initial substrate concentration, *v* is the initial catalytic reaction rate, *V*_max_ is the maximum catalytic reaction rate, *K*_m_ is Michaelis–Menten constant, *E* refers to the total enzyme concentration. All assays were performed in triplicate, and the reported values represent the mean ± standard deviation.

### 2.8. Secondary structure analysis of R783A and R783A@UiO-66-NH_2_

The secondary structures of R783A and R783A@UiO-66-NH_2_ were characterized using Fourier transform infrared spectroscopy. The amide I band (1600–1700 cm^−1^), which reflects protein secondary structural features such as α-helix, β-sheet, β-turn, and random coil, was analyzed for this purpose.^27^ Spectral data were smoothed and baseline-corrected using OMNIC 9.2 software within the 1600–1700 cm^−1^ region. Subsequently, Gaussian deconvolution and second-derivative fitting were performed with PeakFit 4.12 to reduce variance and resolve overlapping peaks. The relative content of each secondary structural component was calculated.

### 2.9. Biocatalytic application and recycling of R783A@UiO-66-NH_2_

The catalytic performance of R783A@UiO-66-NH_2_ in the bioconversion of icariin to icariside I was evaluated under optimal reaction conditions. Reactions were carried out in 2 mL Tris–HCl buffer (100 mM, pH 8.0) containing 5 mM icariin and 0.5 U per mL R783A@UiO-66-NH_2_, incubated at 60 °C with shaking at 180 rpm. Time-course samples were collected at regular intervals, and the conversion of icariin and formation of icariside I were quantified by high-performance liquid chromatography (HPLC). The conversion rate was calculated as the percentage of substrate consumed, while the yield of icariside I was expressed relative to the theoretical maximum based on the initial icariin concentration. For comparison, the amount of free enzyme used was equivalent to that of the immobilized form.

The recyclability of R783A@UiO-66-NH_2_ was assessed over consecutive reaction cycles. After each cycle, the immobilized enzyme was recovered by centrifugation, and the supernatant was filtered and analyzed by HPLC. The recovered catalyst was washed three times with fresh Tris–HCl buffer (100 mM, pH 8.0) to remove residual substrate and products, and then resuspended in fresh reaction medium containing 5 mM icariin for the subsequent cycle. This process was repeated for multiple cycles, with enzyme performance evaluated by monitoring substrate conversion and product yield in each cycle. The protein concentration in the supernatant was determined by the Bradford assay to monitor enzyme leaching from the immobilized biocatalyst. All the experiments were conducted at least in triplicate.

Quantitative analysis of flavonoid compounds was performed using a ZORBAX Eclipse XDB-C18 column (4.6 × 250 mm, 5 µm; Agilent, USA) on a Shimadzu LC-16AT reversed-phase HPLC system (Shimadzu, Japan). The mobile phase consisted of acetonitrile (A) and 0.1% formic acid in water (B) at a flow rate of 1.0 mL min^−1^. The gradient elution program was as follows: 0–12 min, 28% A; 12–17 min, 28% A to 90% A; 17–18 min, 90% A; 18–22 min, 90% A to 28% A; 22–28 min, 28% A. The column temperature was maintained at 35 °C, the injection volume was 10 µL, and detection was carried out at 270 nm.

### 2.10. Statistical analysis

All experiments were performed with three independent replicates (*n* = 3), and data are presented as mean ± standard deviation (mean ± SD). Between-group comparisons were performed using two-way ANOVA with enzyme form (free *vs.* immobilized) and treatment condition as the two factors, followed by Tukey's HSD *post hoc* test. The significance threshold was set at *p* < 0.05.

## 3. Results and discussion

### 3.1. Expression and purification of recombinant α-l-rhamnosidase R783A

The recombinant α-l-rhamnosidase mutant R783A was successfully expressed in *Escherichia coli* BL21(DE3) and purified using Ni-NTA affinity chromatography. The coding sequence of the R783A mutant comprises 2763 nucleotides, encoding a protein of 921 amino acids with a His-tag and a calculated molecular weight of approximately 102 kDa. SDS-PAGE analysis of the crude His-tagged fusion protein revealed a band at the expected molecular weight, indicating efficient expression of soluble R783A. The target protein was subsequently purified and enriched, confirming the successful isolation of the recombinant α-l-rhamnosidase mutant R783A (Fig. 1). The molecular weight of the enzyme was approximately 102 kDa without the other bands, which is consistent with the theoretical molecular weight. The R783A mutant was selected as the enzyme for immobilization because it exhibited higher catalytic activity and improved thermal stability compared with the wild-type (WT) enzyme (Fig. S1). These advantages made R783A a more suitable candidate for immobilization, where the primary goal is to further enhance its reusability and operational stability while retaining its superior catalytic performance.

**Fig. 1:**
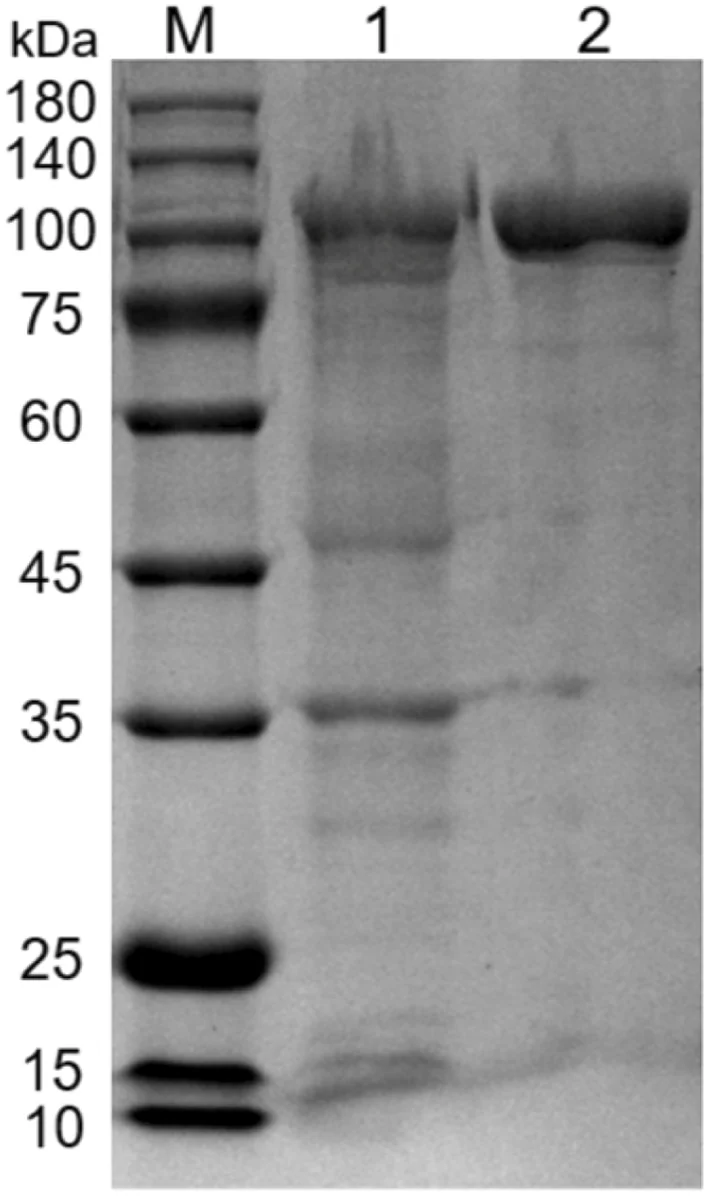
SDS-PAGE analysis of α-l-rhamnosidase mutant R783A; lane M, molecular weight standards; lane 1, the whole-cell lysate after induction; lane 2, the purified R783A protein.

### 3.2. Preparation of R783A@UiO-66-NH_2_ and optimization of immobilization

The preparation process of R783A@UiO-66-NH_2_ was illustrated in Scheme 1. To establish an efficient immobilization strategy, the effects of UiO-66-NH_2_ mass and immobilization time on the enzyme loading and activity recovery were investigated. The mass ratio between enzyme and carrier is a critical factor affecting enzyme loading and catalytic performance.^28^ Therefore, a fixed amount of R783A was immobilized onto different amounts of UiO-66-NH_2_ to evaluate the influence of carrier dosage on immobilization efficiency. As shown in Fig. 2(a), enzyme loading decreased with increasing UiO-66-NH_2_ dosage, whereas the activity recovery exhibited the opposite trend and increased significantly. This phenomenon may be attributed to the lower enzyme density on the carrier surface at higher carrier dosages, which reduced steric hindrance among neighboring enzyme molecules and improved substrate accessibility to the catalytic sites. Similar trends have been reported in previous studies.^26,29^ Notably, the activity recovery reached approximately 88.7% and subsequently remained nearly constant with further increases in UiO-66-NH_2_ dosage. Since physical adsorption is simple, mild, and requires no chemical crosslinking reagents, it helped preserve the native conformation of the enzyme. In addition, immobilization on UiO-66-NH_2_ may provide a more suitable microenvironment for catalysis.^30,31^ Considering both catalytic performance and carrier utilization, 20 mg of UiO-66-NH_2_ was selected as the optimal carrier dosage for subsequent experiments. Immobilization time is another important parameter influencing the interaction between the enzyme and the UiO-66-NH_2_ carrier. As shown in Fig. 2(b), both enzyme loading and activity recovery increased with prolonged incubation time during the initial stage, indicating progressive adsorption of R783A onto the UiO-66-NH_2_ surface. After 3 h, no significant increase was observed, suggesting that adsorption equilibrium had been established between the enzyme and the carrier. Therefore, an immobilization time of 3 h was selected as the optimal condition for the preparation of R783A@UiO-66-NH_2_. Compared with the encapsulation and magnetic-MOF immobilization methods previously used in our group,^15,19^ the present physical adsorption strategy offers several practical advantages, including a simple and facile preparation procedure and a high enzyme loading capacity.

**Scheme 1:**
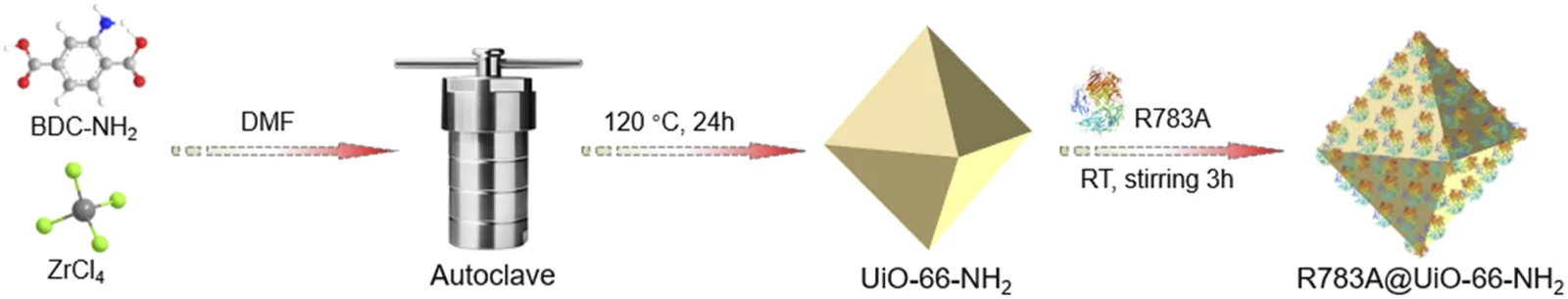
Synthesis process of the R783A@UiO-66-NH_2_.

**Fig. 2:**
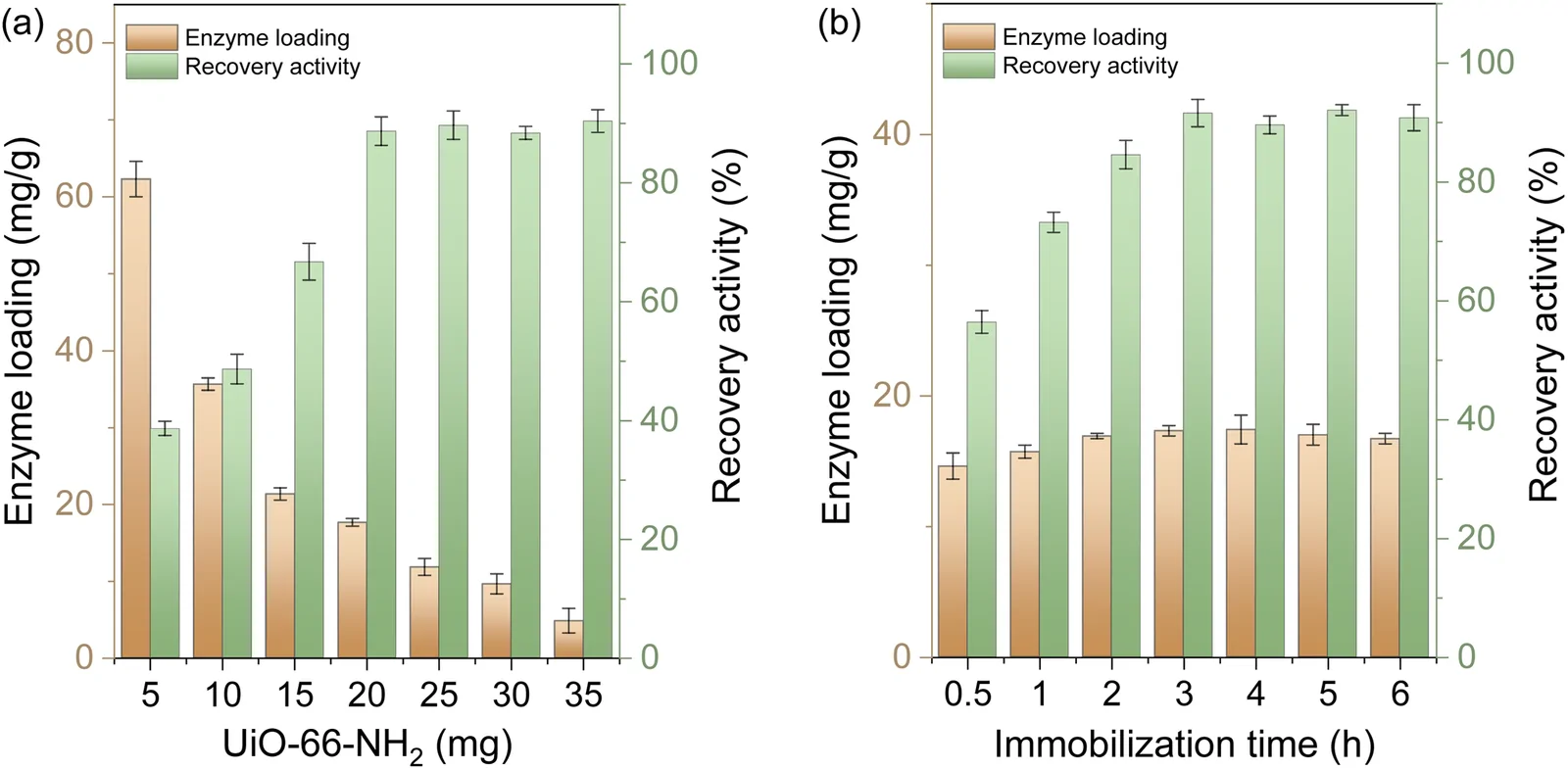
Effects of UiO-66-NH_2_ mass (a) and immobilization time (b) on the immobilization efficiency. Error bars represent means ± SD (*n* = 3).

### 3.3. Characterization

The SEM images of the surface morphology of UiO-66-NH_2_ and R783A@UiO-66-NH_2_ were presented in Fig. 3(a) and (b), respectively. UiO-66-NH_2_ exhibited a uniform and regular morphology with an average particle size of approximately 200 nm, and the resulting nanostructures exhibited smooth surfaces. Following immobilization of R783A onto UiO-66-NH_2_, the particle surface became rougher, while the overall particle size of R783A@UiO-66-NH_2_ remained largely unchanged compared with that of UiO-66-NH_2_ (Fig. 3(b)). Additionally, EDS mapping analysis showed a homogeneous distribution of Zr, C, N, O, and S elements in R783A@UiO-66-NH_2_ (Fig. 3(c)). Since N was already present in the UiO-66-NH_2_ framework, the N signal was not enzyme-specific; however, the detection of S, which was absent from the bare support, provided supporting evidence for the presence of R783A on the UiO-66-NH_2_ surface.

**Fig. 3:**
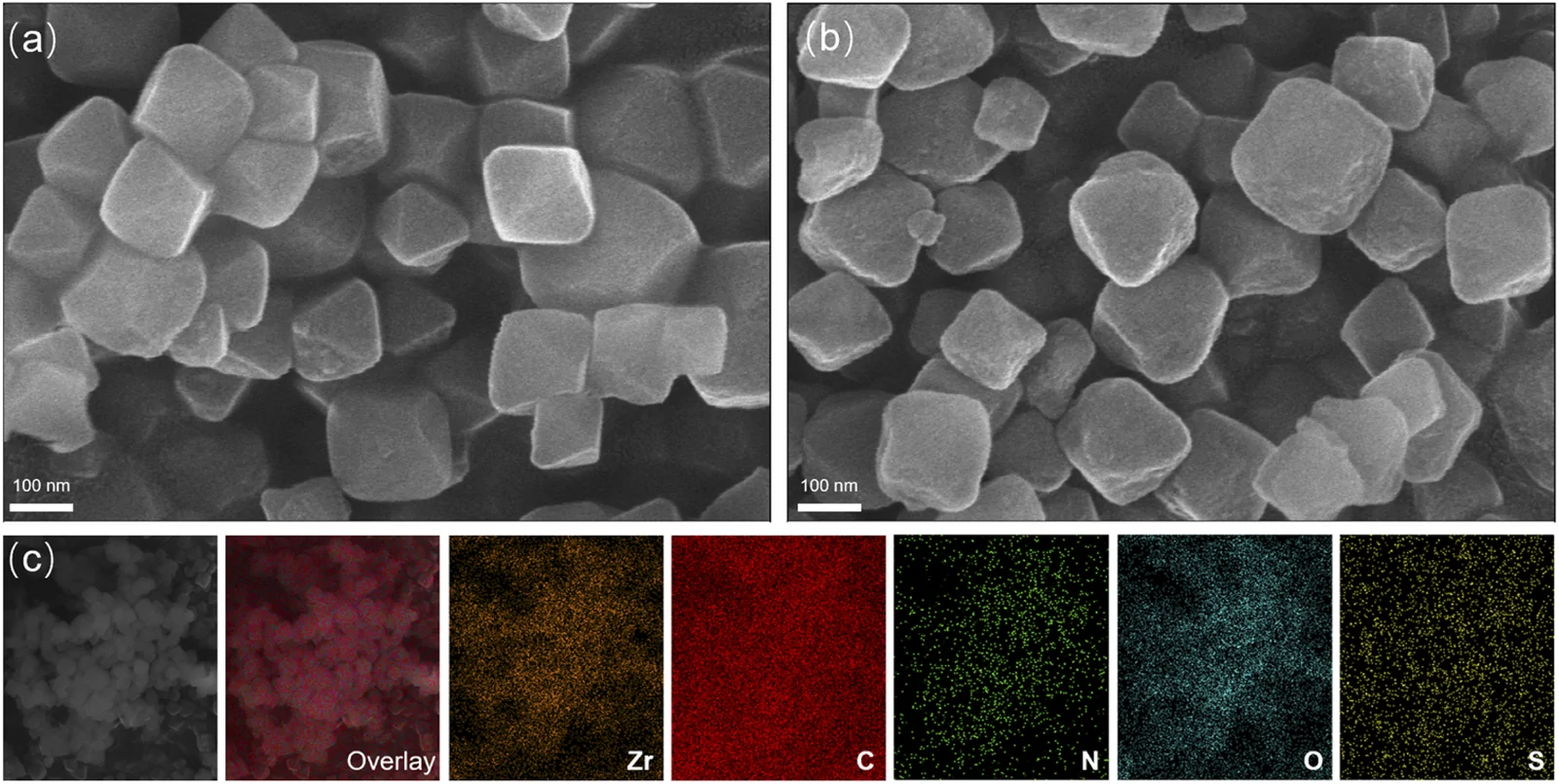
SEM images of the UiO-66-NH_2_ (a) and R783A@UiO-66-NH_2_ (b), and SEM image with corresponding EDS mapping for R783A@UiO-66-NH_2_ (c).

The crystal structures of UiO-66-NH_2_ and R783A@UiO-66-NH_2_ were investigated by XRD (Fig. 4(a)). The characteristic diffraction peaks of UiO-66-NH_2_ at 2*θ* = 7.4°, 8.5°, and 25.8° were consistent with those reported previously,^32,33^ confirming the successful synthesis of the material. Notably, these diffraction peaks were well preserved after R783A immobilization, with no significant peak shift or disappearance observed. This result indicated that the immobilization process exerted a negligible effect on the crystalline structure of UiO-66-NH_2_ and that the framework integrity was maintained. FT-IR analysis was conducted to verify the synthesis of UiO-66-NH_2_ and the immobilization of R783A (Fig. 4(b)). For UiO-66-NH_2_, the characteristic bands at 3431 and 3373 cm^−1^ correspond to the stretching vibrations of free amino groups in UiO-66-NH_2_.^34^ While the peaks at 1401 and 1575 cm^−1^ correspond to the symmetric and asymmetric stretching vibrations of coordinated carboxylate groups (–COO^−^), confirming the formation of Zr–carboxylate coordination bonds.^35^ The characteristic bands at 600–800 cm^−1^ are attributed to Zr–O bending vibrations, further evidencing the successful construction of the UiO-66-NH_2_ framework.^36^ After enzyme immobilization, the FT-IR spectrum of R783A@UiO-66-NH_2_ retained all characteristic peaks of the pristine UiO-66-NH_2_, indicating that the framework structure remained intact during the immobilization process. Meanwhile, a new absorption band appeared at 1025 cm^−1^, which can be attributed to protein-related vibrational modes associated with the secondary structure of R783A. Furthermore, the broadening and increased intensity of the band in the 3200–3500 cm^−1^ region, together with the appearance of characteristic amide I (1600–1700 cm^−1^) and amide II (1500–1560 cm^−1^) bands, provide additional evidence for the successful immobilization of R783A on the UiO-66-NH_2_ matrix. TGA was performed to evaluate the thermal stability of the support material and enzyme loading (Fig. 4(c)). The TGA profile of UiO-66-NH_2_ exhibited three distinct weight-loss stages. The initial weight loss below 100 °C was attributed to the removal of physically adsorbed water molecules and residual solvents confined within the porous structure. The second weight-loss stage, occurring between 100 and 250 °C, was associated with the dehydroxylation of the organic linkers and partial cleavage of coordination bonds within the framework. A substantial weight loss above 350 °C was observed, corresponding to the collapse and decomposition of the UiO-66-NH_2_ framework.^37^ Compared with pristine UiO-66-NH_2_, R783A@UiO-66-NH_2_ exhibited a noticeably greater weight loss in the range of 30–150 °C. This additional mass loss is mainly attributed to the removal of adsorbed water and residual solvents, with a possible contribution from the thermal desorption and decomposition of weakly adsorbed R783A molecules.

**Fig. 4:**
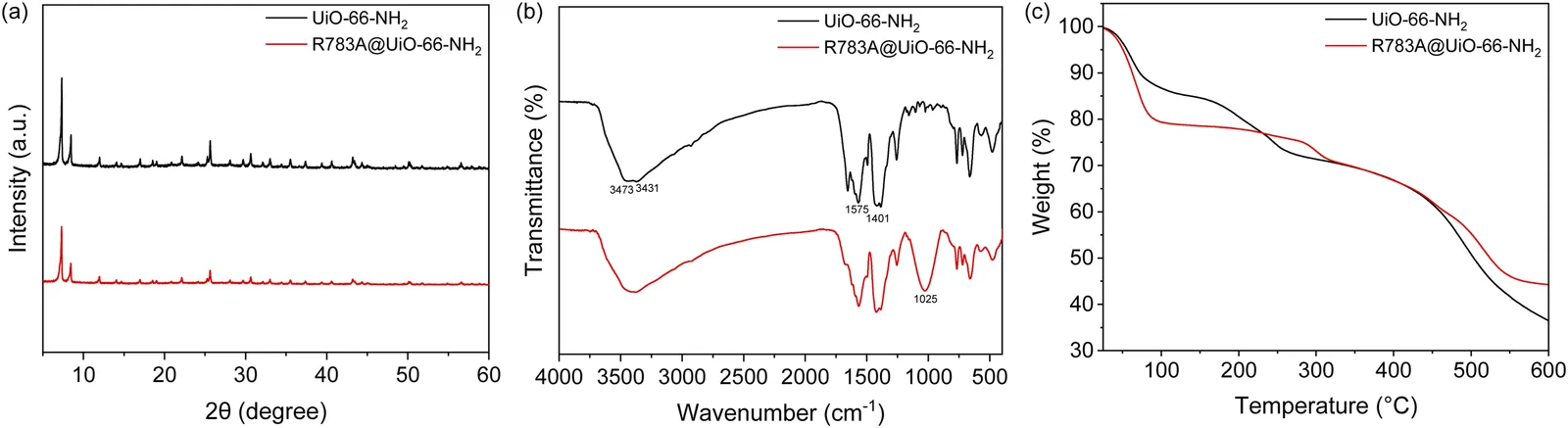
XRD patterns (a), FTIR spectra (b) and TGA curves (c) of UiO-66-NH_2_ and R783A@UiO-66-NH_2_.

### 3.4. Enzymatic properties of R783A@UiO-66-NH_2_

#### 3.4.1. Condition optimization and stability assessment

To comprehensively evaluate the effects of immobilization on the enzymatic properties of R783A, the pH and temperature profiles of free R783A and immobilized R783A@UiO-66-NH_2_ were systematically investigated (Fig. 5). As shown in Fig. 5(a), the activities of both free R783A and R783A@UiO-66-NH_2_ exhibited a similar trend, increasing with rising pH and reaching a maximum at pH 8.0, followed by a gradual decrease under more alkaline conditions. Nevertheless, R783A@UiO-66-NH_2_ displayed slightly higher activity retention across the tested pH range 6–9 compared with free R783A. The improved pH tolerance can be attributed to the MOF microenvironment, where amino groups and surface charge regulate local proton concentration and electrostatic interactions around the catalytic center, mitigating pH-induced structural perturbations.^34,38^ The pH stability of free R783A and R783A@UiO-66-NH_2_ was assessed by measuring residual activity after incubation in buffers of varying pH for 2 h (Fig. 5(c)). Although only minor differences were observed under acidic conditions, R783A@UiO-66-NH_2_ consistently exhibited higher residual activity than free R783A across the pH range of 5.0–9.0, indicating that immobilization on the UiO-66-NH_2_ framework enhanced resistance to pH-induced deactivation. The effect of temperature on the catalytic activity of free R783A and R783A@UiO-66-NH_2_ were presented in Fig. 5(b). Free R783A exhibited its maximum activity at 70 °C, whereas R783A@UiO-66-NH_2_ showed a shifted optimum temperature of 80 °C. The immobilized enzyme also retained more than 82% relative activity over a broad temperature range (60–90 °C), indicating that it remained catalytically functional at elevated temperatures. This shift indicated that immobilization altered the temperature-activity profile of the enzyme, possibly by providing a microenvironment that helps preserve the active conformation at higher temperatures.^39^ Thermal stability is of critical importance for applications of enzymes, as high operating temperatures are generally preferred due to inhibited microbial growth and improved solubility of poorly water-soluble substrates such as naringin, rutin, and hesperidin.^40^ As shown in Fig. 5(d), both free R783A and R783A@UiO-66-NH_2_ retained more than 90% of their initial activity after incubation at 60 °C for 2 h. This indicated that both enzyme forms were thermally stable at 60 °C, with no significant difference observed between them under this condition. These results suggested that the immobilized enzyme preserves the intrinsic thermal stability of the free enzyme, while the potential stabilizing effect of immobilization may become more evident at higher temperatures where denaturation is more severe.

**Fig. 5:**
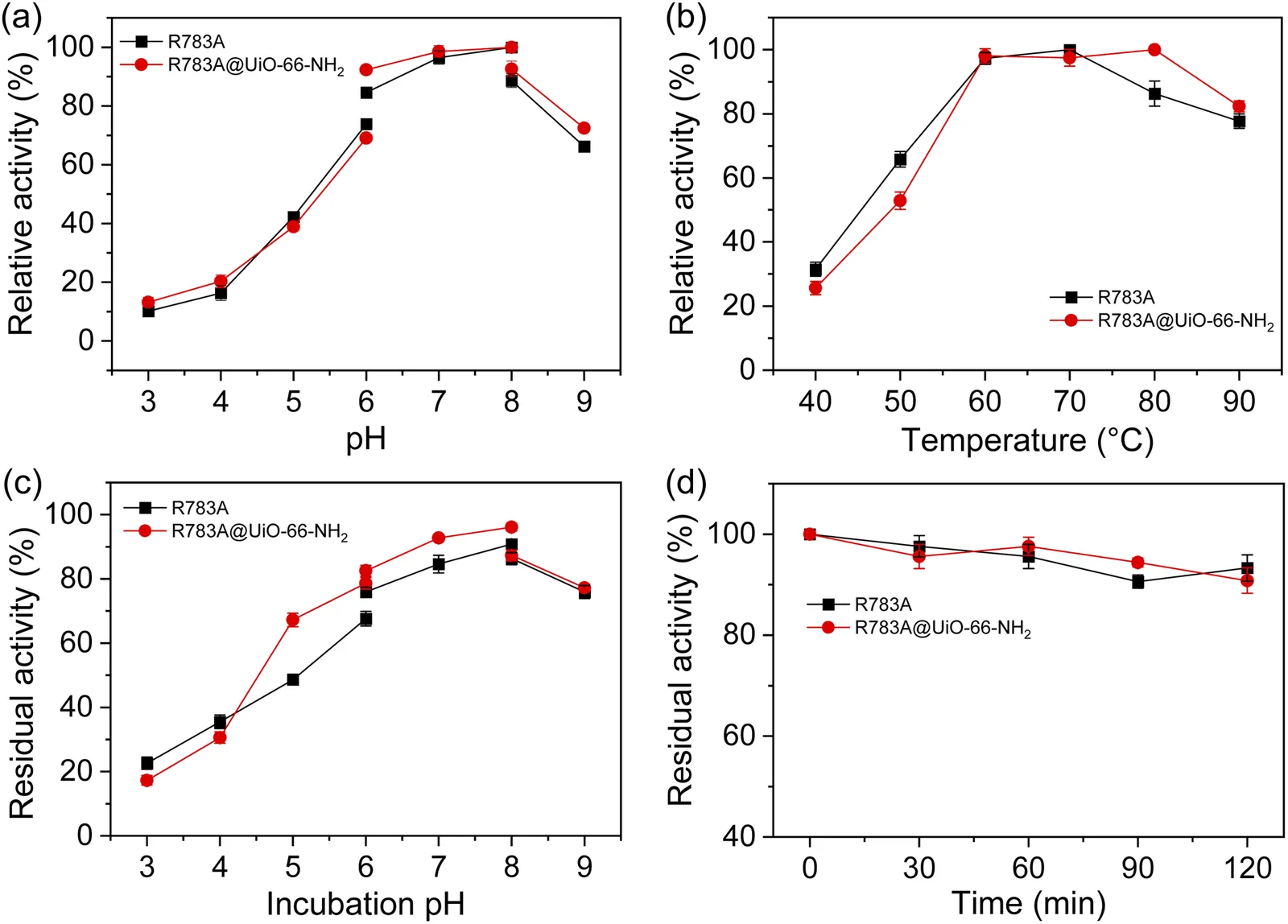
Effects of pH and temperature on the activity and stability of the free R783A and R783A@UiO-66-NH_2_. The optimal pH (a), temperature (b), pH stability (c) and thermostability at 60 °C (d) of the free R783A and R783A@UiO-66-NH_2_. Error bars represent means ± SD (*n* = 3).

Storage stability is a critical parameter for the practical application of biocatalysts, as minimal activity loss over time is highly desirable. As depicted in Fig. S2, both free R783A and R783A@UiO-66-NH_2_ retained the majority of their catalytic activity throughout the 30-day storage period, with the immobilized enzyme consistently exhibiting higher residual activity. After 30 days, R783A@UiO-66-NH_2_ maintained 88.6% of its initial activity, whereas free R783A retained 81.1%. This enhanced storage stability may be attributed to the protective microenvironment provided by the MOF support, which effectively suppresses enzyme aggregation and preserves the native protein conformation.^41^ Similar observations have been reported by Yuan *et al.*, who demonstrated that UiO-66-NH_2_-immobilized β-glucosidase retained 72% of its initial activity after 30 days of storage.^42^

#### 3.4.2. Effects of metal ions and organic reagents on enzyme activity

The effects of metal ions on the activities of free R783A and R783A@UiO-66-NH_2_ were presented in Fig. 6(a). The immobilized enzyme exhibited higher relative activities than the free enzyme in the presence of most tested metal ions. Notably, the relative activities of R783A@UiO-66-NH_2_ in reaction systems containing 1 mM Fe^2+^ and Fe^3+^ were significantly higher than those of free R783A, indicating metal-dependent activation of R783A by these ions. For the other metal ions tested, immobilization generally resulted in only marginal improvements in enzyme activity. However, the tolerance of R783A@UiO-66-NH_2_ toward Cu^2+^ and Zn^2+^, which are known inhibitory metal ions for many glycosidases, was a slight enhanced compared with that of the free enzyme. This improvement was likely attributable to the metal-ion adsorption capacity of Zr-based MOFs, whose exposed surface, resulting from the low enzyme loading ratio, may sequester metal ions and thereby mitigate their inhibitory effects,^43^ as supported by the metal-ion concentration measurements before and after exposure to R783A@UiO-66-NH_2_ (Table S1). In addition, DTT and EDTA showed no significant effects on the activities of either free or immobilized R783A. The enzyme retained activity under the tested DTT and EDTA conditions, indicating that its activity is not strongly dependent on readily reducible disulfide bonds or on chelatable metal ions under these conditions, without ruling out such roles in enzyme structure or catalysis.

**Fig. 6:**
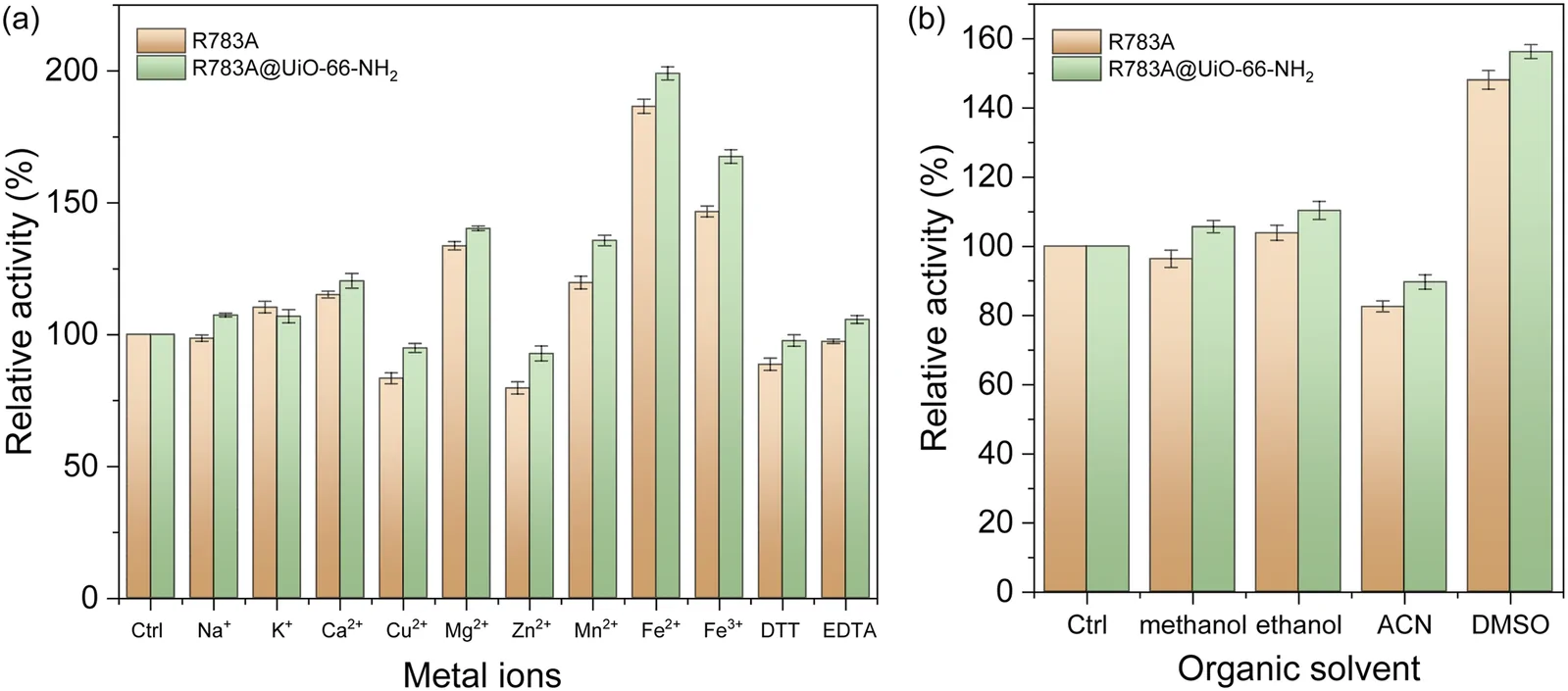
Effects of metal ions (a) and organic reagents (b) on the activity of the free R783A and R783A@UiO-66-NH_2_. Error bars represent means ± SD (*n* = 3).

The effects of common organic solvents on the activities of free R783A and R783A@UiO-66-NH_2_ were presented in Fig. 6(b). Methanol and ethanol showed no significant effects on the activities of either enzyme, whereas acetonitrile (ACN) exerted a slight inhibitory effect. In all tested organic solvents, the immobilized enzyme consistently exhibited higher relative activity than the free enzyme. Notably, both enzyme forms showed strong tolerance to DMSO, with relative activities of 148.1% and 156.3%, respectively, which was consistent with our previous report on the wild-type α-l-rhamnosidase exhibiting good activity in the presence of DMSO.^10^ The higher activity of R783A@UiO-66-NH_2_ in the presence of organic solvents was beneficial for improving the catalytic efficiency toward poorly water-soluble flavonoid substrates.

#### 3.4.3. Kinetic parameter analysis

To elucidate the effect of immobilization on the catalytic behavior of R783A, the apparent kinetic parameters of both free R783A and immobilized R783A@UiO-66-NH_2_ were determined based on Michaelis–Menten kinetics (Table 1 and Fig. S3). The results provided insight into how the UiO-66-NH_2_ microenvironment influences enzyme–substrate interactions and catalytic efficiency. The *V*_max_ of R783A@UiO-66-NH_2_ was slightly higher than that of the free enzyme (2.83 *vs.* 2.74 mM s^−1^), indicating that immobilization did not impose significant mass-transfer limitations and may even create a more favorable catalytic microenvironment for catalysis. Conversely, the *K*_m_ increased from 0.45 to 0.53 mM upon immobilization, reflecting a modest reduction in substrate affinity. This phenomenon was commonly observed in immobilized enzyme systems and may be attributed to diffusion limitations and/or partial steric hindrance imposed by the support matrix.^44,45^ In agreement with this trend, Jiang *et al.* reported that laccase immobilized on a Cu-based MOF exhibited a *K*_m_ of 0.2737 mM, which was higher than that of the free enzyme (0.0763 mM).^46^ Despite the decreased apparent affinity, the *k*_cat_ of R783A@UiO-66-NH_2_ (23.06 s^−1^) was higher than that of the free enzyme (18.37 s^−1^), suggesting an enhancement in catalytic turnover once the substrate was bound to the active site. As a result, the catalytic efficiency *k*_cat_/*K*_m_ of the R783A@UiO-66-NH_2_ was slightly improved relative to that of the free enzyme, indicating an overall beneficial effect of UiO-66-NH_2_ immobilization on catalytic performance.

**Table 1:** Apparent kinetic parameters of free R783A and R783A@UiO-66-NH_2_^a^

Enzyme	*V*_max_ (mM s^−1^)	*K*_m_ (mM)	*k*_cat_ (s^−1^)	*k*_cat_/*K*_m_ (s^−1^ mM^−1^)
R783A	2.74 ± 0.027	0.45 ± 0.070	18.37 ± 2.2	40.8 ± 1.3
R783A@UiO-66-NH_2_	2.83 ± 0.028*	0.53 ± 0.031*	23.06 ± 0.7**	43.5 ± 1.1*

a The experimental data are presented as mean ± SD (*n* = 3). Statistical differences were analyzed using independent samples *t*-test. ** indicates highly significant difference (*P* < 0.01), and * indicates significant difference (*P* < 0.05).

### 3.5. Secondary structure changes of R783A@UiO-66-NH_2_

To investigate the effect of immobilization on the secondary structure of R783A, the amide I region of the FTIR spectra was deconvoluted and curve-fitted, and the results were presented in Fig. 7 and Table 2. Immobilized R783A exhibited increased proportions of β-sheet and β-turn structures relative to the free enzyme, which may reflect local conformational adjustments at the enzyme–support interface. These changes were accompanied by slight decreases in α-helix and random coil contents. The reduction in α-helix content may suggest that partial conformational rearrangement occurred during immobilization. Although β-sheet structures are relatively susceptible to conformational transitions at elevated temperatures because of their intrinsic flexibility,^47^ the thermal stability of R783A@UiO-66-NH_2_ at 60 °C was comparable to that of the free enzyme, suggesting that the slight changes in β-sheet structure have little effect on its thermal stability. This interpretation appears consistent with the improved storage stability (Fig. S2) and enhanced tolerance toward metal ions and organic reagents (Fig. 6) exhibited by R783A@UiO-66-NH_2_. It should be noted that these changes are relatively small, and further investigation in future studies would be valuable.

**Fig. 7:**
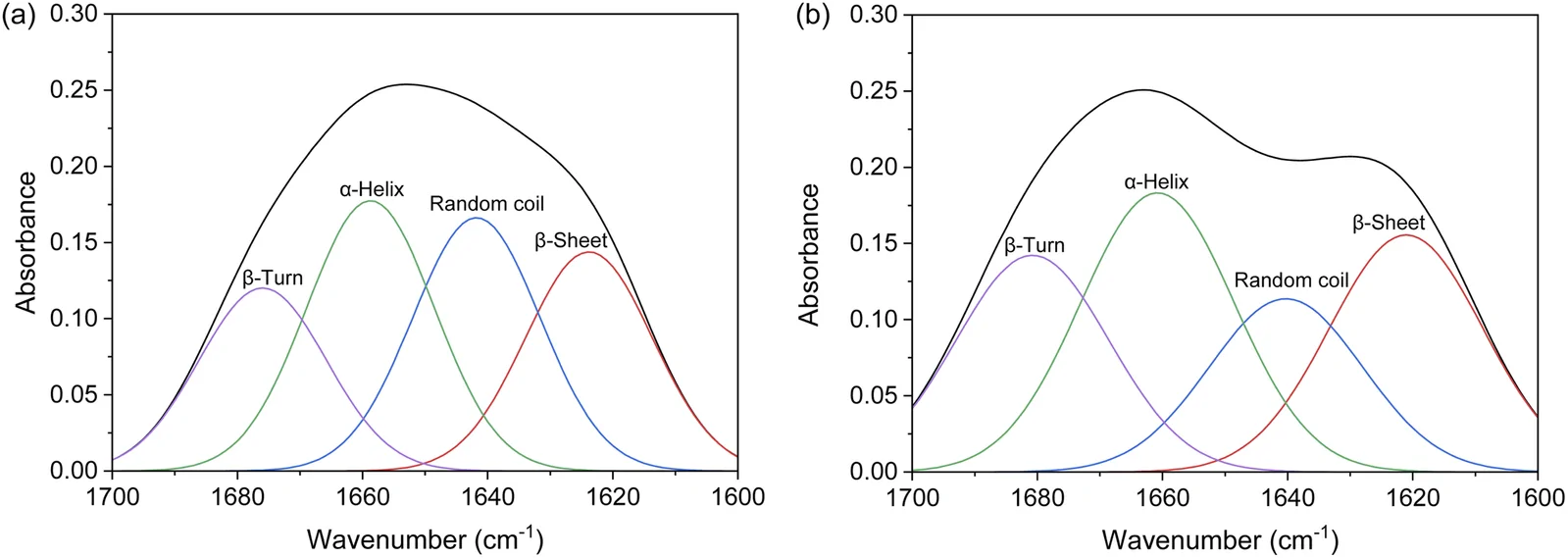
Fitting of IR spectra in the amide I region for free R783A (a) and R783A@UiO-66-NH_2_ (b).

**Table 2:** Secondary structures of free R783A and R783A@UiO-66-NH_2_ within the amide I region

Enzymes	α-Helix (%)	β-Sheet (%)	β-Turn (%)	Random coil (%)
R783A	31.9	23.6	18.3	26.2
R783A@UiO-66-NH_2_	29.3	26.7	24.9	19.1

### 3.6. Biocatalytic application of R783A@UiO-66-NH_2_ for production of icariside I from icariin

To comprehensively evaluate the catalytic performance of the immobilized system, we first examined the hydrolytic activities of free R783A and R783A@UiO-66-NH_2_. HPLC analysis demonstrated that both enzyme forms effectively catalyzed the stepwise hydrolysis of icariin through the selective cleavage of the rhamnose moiety at the C-3 position, ultimately yielding icariside I as the major final product (Fig. S4). The time-course analysis of icariin biotransformation was shown in Fig. 8(a). Upon addition of 5 mM icariin and incubation at 60 °C, free R783A exhibited a slightly higher initial reaction rate than immobilized R783A@UiO-66-NH_2_ within the first hour. This was mainly attributed to the direct accessibility of free enzyme to the substrate, which minimized mass transfer limitations at the early stage of the reaction.^46^ However, after 1 h, the catalytic activity of free R783A gradually declined, whereas R783A@UiO-66-NH_2_ maintained sustained catalytic performance. As a result, R783A@UiO-66-NH_2_ achieved 99.8% icariin conversion and 97.6% icariside I yield within 5 h, corresponding to a space-time yield of 0.52 g L^−1^ h^−1^. While free R783A required 6 h to reach comparable values (99.7% conversion and 96.8% yield). These results indicated that both enzyme forms are highly efficient in the first reaction cycle. Therefore, the principal advantage of immobilization in the first cycle is the shortened reaction time (∼1 h). In addition, this difference may also be associated with the slightly higher catalytic efficiency of the immobilized enzyme, as reflected by the kinetic parameters (Table 1).

**Fig. 8:**
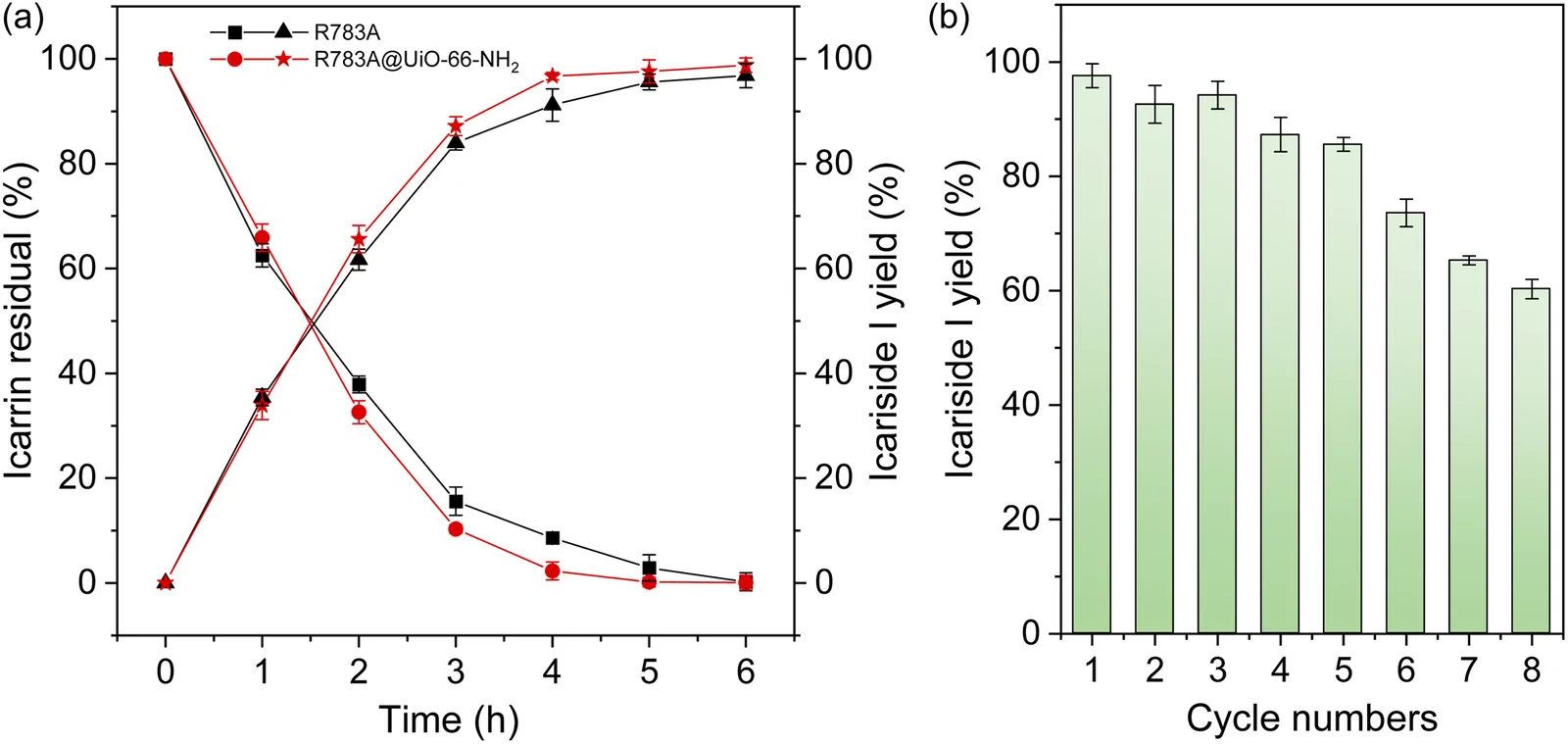
(a) Time-course profiles of icariside I production catalyzed by free R783A and immobilized R783A@UiO-66-NH_2_. (b) Reusability of R783A@UiO-66-NH_2_ during consecutive catalytic cycles. Error bars represent means ± SD (*n* = 3).

Reusability is one of the most important advantages of immobilized enzymes, as it directly affects process economics and industrial feasibility. The ability to recover and reuse biocatalysts not only reduces enzyme consumption and production costs but also facilitates product separation and process operation. Accordingly, the reusability of R783A@UiO-66-NH_2_ was investigated through repeated catalytic cycles, and the results were shown in Fig. 8(b). The immobilized biocatalyst exhibited excellent operational stability during the initial cycles, maintaining an icariside I yield above 90% over the first three consecutive reaction cycles. After five cycles, R783A@UiO-66-NH_2_ still afforded an icariside I yield of 85.6%, indicating good retention of catalytic activity. However, a noticeable decline in catalytic performance was observed from the sixth cycle onward, with the product yield decreasing to 60.3% after the eighth cycle. This gradual loss of activity was primarily attributed to enzyme leaching from the carrier, as confirmed by the protein-leaching measurement (Fig. S5). SEM characterization of the recovered biocatalyst after eight cycles indicated that the structural integrity of the biocatalyst was largely maintained (Fig. S6), although minor structural changes caused by prolonged exposure to reaction conditions may have contributed to a lesser extent. This enzyme leaching was also likely associated with the physical adsorption strategy, in which the relatively weak binding strength between the enzyme and the carrier facilitates gradual desorption during repeated use. In addition, considering potential zirconium leaching, ICP-OES analysis of the reaction supernatant after the 8th cycle revealed only trace levels of zirconium (0.223 mg L^−1^), substantially below the permitted limits for elemental impurities in drug products established by the ICH guideline (Table S2). Nevertheless, R783A@UiO-66-NH_2_ retained more than 60% of its catalytic performance after eight consecutive cycles at 60 °C, demonstrating considerable operational stability under relatively harsh reaction conditions. Notably, the reusability of R783A@UiO-66-NH_2_ compares favorably with that of previously reported enzyme-MOF biocatalysts. For example, β-glucosidase immobilized on UiO-66-NH_2_ afforded a kinsenoside yield of only 27.02% after three reuse cycles.^42^ In comparison, R783A@UiO-66-NH_2_ maintained an icariside I yield of 85.6% after five cycles, highlighting the effectiveness of the Zr-based MOF immobilization strategy and its potential for practical biocatalytic applications.

## 4. Conclusion

In this study, the α-l-rhamnosidase mutant R783A was successfully heterologously expressed, purified, and immobilized onto UiO-66-NH_2_*via* a simple adsorption strategy to construct the biocatalyst R783A@UiO-66-NH_2_. Comprehensive characterization confirmed the successful immobilization of R783A on the UiO-66-NH_2_ support. Adsorption on UiO-66-NH_2_ shifted the activity optimum from 70 to 80 °C. The immobilized enzyme showed improved pH tolerance (6.0–9.0), enhanced storage stability (88.6% residual activity after 30 days *vs.* 81.1% for the free enzyme), and improved resistance to metal ions and organic solvents, while the apparent *k*_cat_/*K*_m_ increased modestly from 40.8 to 43.5 s^−1^ mM^−1^. Importantly, R783A@UiO-66-NH_2_ achieved 99.8% conversion of icariin with a 97.6% yield of icariside I within 5 h, corresponding to a space-time yield of 0.52 g L^−1^ h^−1^. The biocatalyst retained 85.6% and 60.3% product yield after five and eight consecutive reuse cycles at 60 °C, respectively, demonstrating good operational stability and reusability. These findings demonstrate that UiO-66-NH_2_ serves as a highly effective immobilization platform, offering a promising strategy for the efficient biotransformation of flavonoid glycosides and related natural products.

## Conflicts of interest

There are no conflicts of interest to declare.

## Supplementary Material

RA-OLF-D6RA06339J-s001

## Data Availability

The data supporting this article have been included as part of the supplementary information (SI). More details are available upon request. Supplementary information: Fig. S1, relative activities of WT and R783A and thermostability of WT and R783A; Fig. S2, storage stability of the free R783A and R783A@UiO-66-NH_2_ at 4 °C; Table S1, adsorption of Cu^2+^ and Zn^2+^ by R783A@UiO-66-NH_2_; Fig. S3, Michaelis–Menten plots of the free R783A and R783A@UiO-66-NH_2_; Fig. S4, HPLC spectrogram for analysis of icariside I from icariin; Fig. S5, concentration of leached protein in the reaction supernatant over 8 cycles; Fig. S6, SEM images of the R783A@UiO-66-NH_2_ after the 8th catalytic cycle; Table S2, leaching of metal ions from R783A@UiO-66-NH_2_ measured by ICP-OES; Fig. S7, the calibration curves of icariin and icariside I; and Table S3, R783A mutant protein sequence. See DOI: https://doi.org/10.1039/d6ra06339j.

## References

[cit1] Huang Z., Li M., Li A., Wei G., Lin X., Jia L., Xue M., Zhang Y., Dong L. (2026). Ind. Crops Prod..

[cit2] Chen C., Wu M., Lei H., Cao Z., Wu F., Song Y., Zhang C., Qin M., Zhang C., Du R. (2023). ACS Pharmacol. Transl. Sci..

[cit3] Gao Y., Xu G., Ma L., Shi W., Wang Z., Zhan X., Qin N., He T., Guo Y., Niu M. (2021). Cell Commun. Signaling.

[cit4] Hou M., Li H., He T., Hui S., Dai W., Hou X., Zhao J., Zhao J., Wen J., Kan W. (2024). J. Pharm. Pharmacol..

[cit5] Chen G., Huang J., Lei H., Wu F., Chen C., Song Y., Cao Z., Zhang C., Zhang C., Ma Y. (2022). Biomed. Pharmacother..

[cit6] Yue M., Liu Y., Feng X., Cao B., Fei X., Li G., Li C. (2025). J. Pharm. Anal..

[cit7] Ke L., Duan X., Cui J., Song X., Ma W., Zhang W., Liu Y., Fan Y. (2023). Carbohydr. Polym..

[cit8] Ma H., He X., Yang Y., Li M., Hao D., Jia Z. (2011). J. Ethnopharmacol..

[cit9] Dong J., Fernández-Fueyo E., Hollmann F., Paul C. E., Pesic M., Schmidt S., Wang Y., Younes S., Zhang W. (2018). Angew. Chem., Int. Ed..

[cit10] Hu J., Song L., Zhao L., Zheng X., Feng W., Jia H. (2025). Molecules.

[cit11] Hassan M. E., Zhu X., de Souza E. F., Elnashar M. M., Lu F. (2025). Green Chem..

[cit12] Vishal K., Barman S., Senger D. S., Yadav V., Yadav P. K. (2025). Biotechnol. Appl. Biochem..

[cit13] Ban Y., Yang H., Jiang J., Wang C., Lv B., Feng Y. (2024). Int. J. Biol. Macromol..

[cit14] Li L., Gong J., Wang S., Li G., Gao T., Jiang Z., Cheng Y.-S., Ni H., Li Q. (2019). J. Agric. Food Chem..

[cit15] Jia H., Song L., Yan T., Wang L., Hu J. (2026). Ind. Crops Prod..

[cit16] Du Y., Jia X., Zhong L., Jiao Y., Zhang Z., Wang Z., Feng Y., Bilal M., Cui J., Jia S. (2022). Coord. Chem. Rev..

[cit17] Kuang G., Wang Z., Bilal M., Wang Z., Feng Y., Du Y., Cui J. (2025). Aggregate.

[cit18] Patil P. J., Usman M., Dong X., Shah H., Manurkar N., Zhang C., Ali A., Li X. (2026). Crit. Rev. Biotechnol..

[cit19] Jia H., Hu J., Zhao L., Zheng X., Feng W. (2025). Mol. Catal..

[cit20] Wang D., Zheng P., Chen P., Wu D. (2021). Bioresour. Technol..

[cit21] Sun X., Nian L., Qi H., Wang M., Huang D., Cao C. (2025). Green Chem..

[cit22] Li Z., Liu X., Wu Z., Huang X., Long H., Yue J., Cao S., Fan D. (2024). ACS Appl. Mater. Interfaces.

[cit23] Jia H. Y., Zong M. H., Zheng G. W., Li N. (2019). ChemSusChem.

[cit24] Bradford M. M. (1976). Anal. Biochem..

[cit25] Kandiah M., Nilsen M. H., Usseglio S., Jakobsen S., Olsbye U., Tilset M., Larabi C., Quadrelli E. A., Bonino F., Lillerud K. P. (2010). Chem. Mater..

[cit26] Li N., Xia H., Liu S., Teng J., Jiang Y. (2023). Ind. Crops Prod..

[cit27] Barth A. (2007). Biochim. Biophys. Acta.

[cit28] Zhang D.-H., Yuwen L.-X., Peng L.-J. (2013). J. Chem..

[cit29] Jiang Y., Hu X., Mei Y., Li X., Chen S., Yuan J., Wang Y., Tao R., Si J., Xu Z. (2024). Int. J. Biol. Macromol..

[cit30] Wang B., Wang Z., Chen M., Du Y., Li N., Chai Y., Wang L., Zhang Y., Liu Z., Guo C. (2024). ACS Appl. Mater. Interfaces.

[cit31] Shen X., Du Y., Du Z., Tang X., Li P., Cheng J., Yan R., Cui J. (2023). Mater. Today Chem..

[cit32] Fang X., Wu S., Wu Y., Yang W., Li Y., He J., Hong P., Nie M., Xie C., Wu Z. (2020). Appl. Surf. Sci..

[cit33] Mulik N., Bokade V. (2022). Mol. Catal..

[cit34] Cao S.-L., Yue D.-M., Li X.-H., Smith T. J., Li N., Zong M.-H., Wu H., Ma Y.-Z., Lou W.-Y. (2016). ACS Sustainable Chem. Eng..

[cit35] Fiaz M., Athar M., Rani S., Najam-ul-Haq M., Farid M. A. (2020). Mater. Chem. Phys..

[cit36] Askari S., Jafarzadeh M., Christensen D. B., Kegnæs S. (2020). Catal. Lett..

[cit37] Chen J., Sun B., Sun C., Zhang P., Xu W., Liu Y., Xiong B., Tang K. (2020). Sep. Purif. Technol..

[cit38] Hassabo A. A., Mousa A. M., Abdel-Gawad H., Selim M. H., Abdelhameed R. M. (2019). J. Mater. Chem. B.

[cit39] Peng L., Tan W., Lu Y., Yao A., Zheng D., Li L., Xiao J., Li L., Li Q., Zhou S. F. (2022). ChemCatChem.

[cit40] Li L., Liao H., Yang Y., Gong J., Liu J., Jiang Z., Zhu Y., Xiao A., Ni H. (2018). Int. J. Biol. Macromol..

[cit41] Xu X., Wan J., Sun J., Wu L., Lei J. (2025). Small Sci..

[cit42] Yuan X., Liao J.-H., Du G.-J., Hou Y., Hu S.-Q. (2024). Int. J. Biol. Macromol..

[cit43] Li Y.-H., Wang C.-C., Zeng X., Sun X.-Z., Zhao C., Fu H., Wang P. (2022). Chem. Eng. J..

[cit44] Rodrigues R. C., Ortiz C., Berenguer-Murcia Á., Torres R., Fernández-Lafuente R. (2013). Chem. Soc. Rev..

[cit45] Bolivar J. M., Woodley J. M., Fernandez-Lafuente R. (2022). Chem. Soc. Rev..

[cit46] Jiang S., Ren D., Wang Z., Zhang S., Zhang X., Chen W. (2022). Ecotoxicol. Environ. Saf..

[cit47] Ma B., Liu D., Zheng M., Wang Z., Zhang D., Jian Y., Ma J., Fan Y., Chen Y., Gao Y. (2024). JACS Au.

