# Supplementary material for: Valorization of icariin via α-l-rhamnosidase mutant immobilized on UiO-66-NH2: an efficient and recyclable biocatalytic system for icariside I production
Source: RSC Adv. 2026 Oct 12;16(47):62482–93. doi: 10.1039/d6ra06339j (PMC13657089; doi:10.1039/d6ra06339j)
Supplement: Supplementary file 1 [file RA-016-D6RA06339J-s001.pdf]

Supplementary Information (SI) for RSC Advances.
This journal is © The Royal Society of Chemistry 2026

Valorization of icariin via α-L-rhamnosidase mutant immobilized on UiO-66-NH_2: An efficient and recyclable biocatalytic system for icariside I production

Haoyu Jia[a,*], Tong Yan[a], Luran Wang[a], Lingling Song[a]

[a] School of Pharmacy, Henan University of Chinese Medicine, Zhengzhou, 450046, China

* Corresponding author.

E-mail: jiahaoyu@hactcm.edu.cn (H. Jia)

Fig. S1. Relative activities (a) of WT and R783A and thermostability (b) of WT and R783A.

Fig. S2. Storage stability of the free R783A and R783A@UiO-66-NH_2 at 4 °C.

Table S1. Adsorption of Cu^{2+} and Zn^{2+} by R783A@UiO-66-NH_2.

Fig. S3. Michaelis-Menten plots of the free R783A (a) and R783A@UiO-66-NH_2 (b).

Fig. S4. HPLC Spectrogram for analysis of icariside I from icariin.

Fig. S5 Concentration of leached protein in the reaction supernatant over 8 cycles.

Fig. S6 SEM images of the R783A@UiO-66-NH_2 after the 8th catalytic cycle.

Table S2. Leaching of metal ions from R783A@UiO-66-NH_2 measured by ICP-OES.

Fig. S7 The calibration curves of icarrin and icariside I.

Table S3 R783A mutant protein sequence.

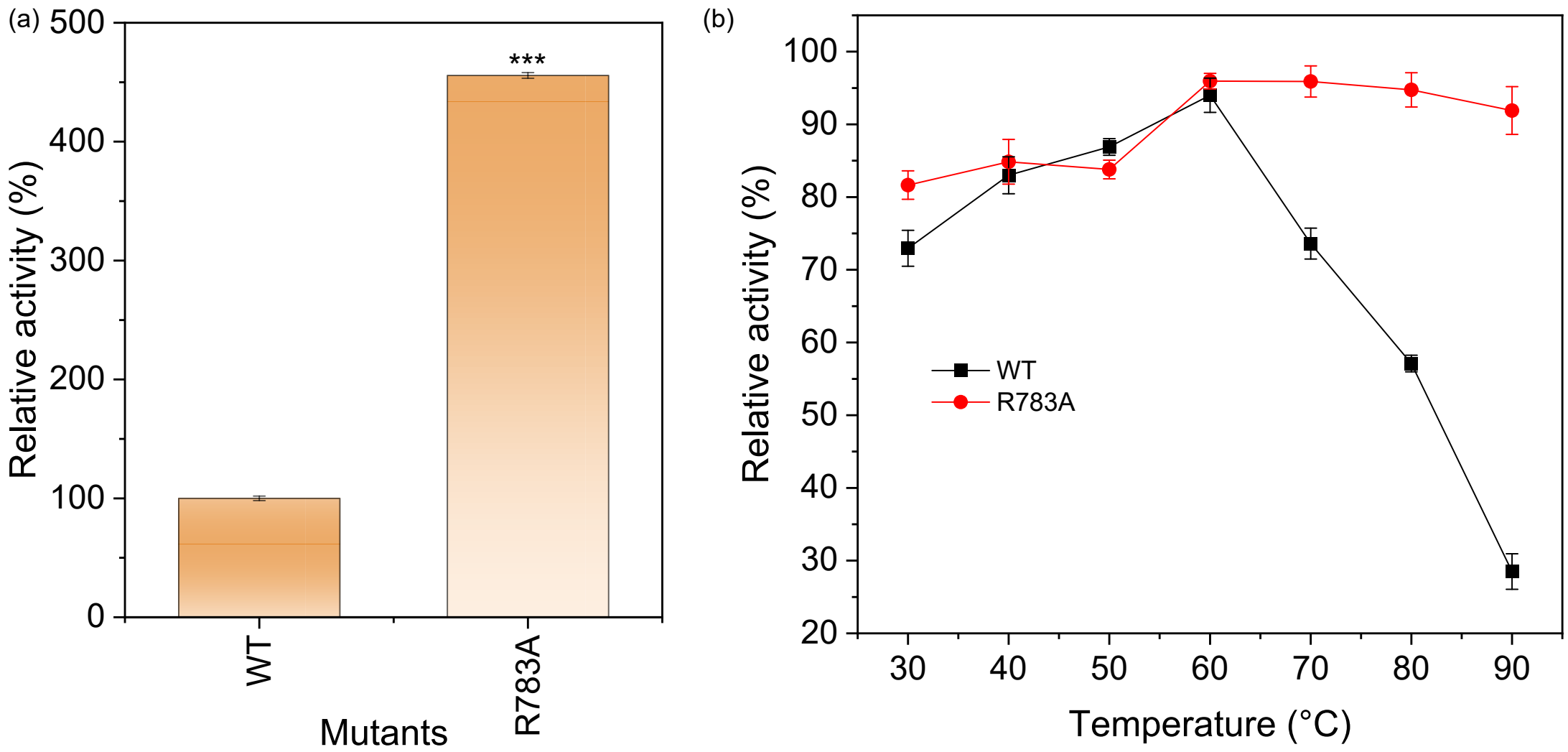

Fig. S1 Relative activities (a) of WT and R783A and thermostability (b) of WT and R783A. Data are presented as mean ± SD from three independent experiments. The enzymatic activity of the WT was defined as 100% and used as the control. Statistical significance was determined using Student's t-test by comparison with the WT. Significance levels are indicated as follows: ns, not significant; *p< 0.05; **p < 0.01; ***p < 0.001

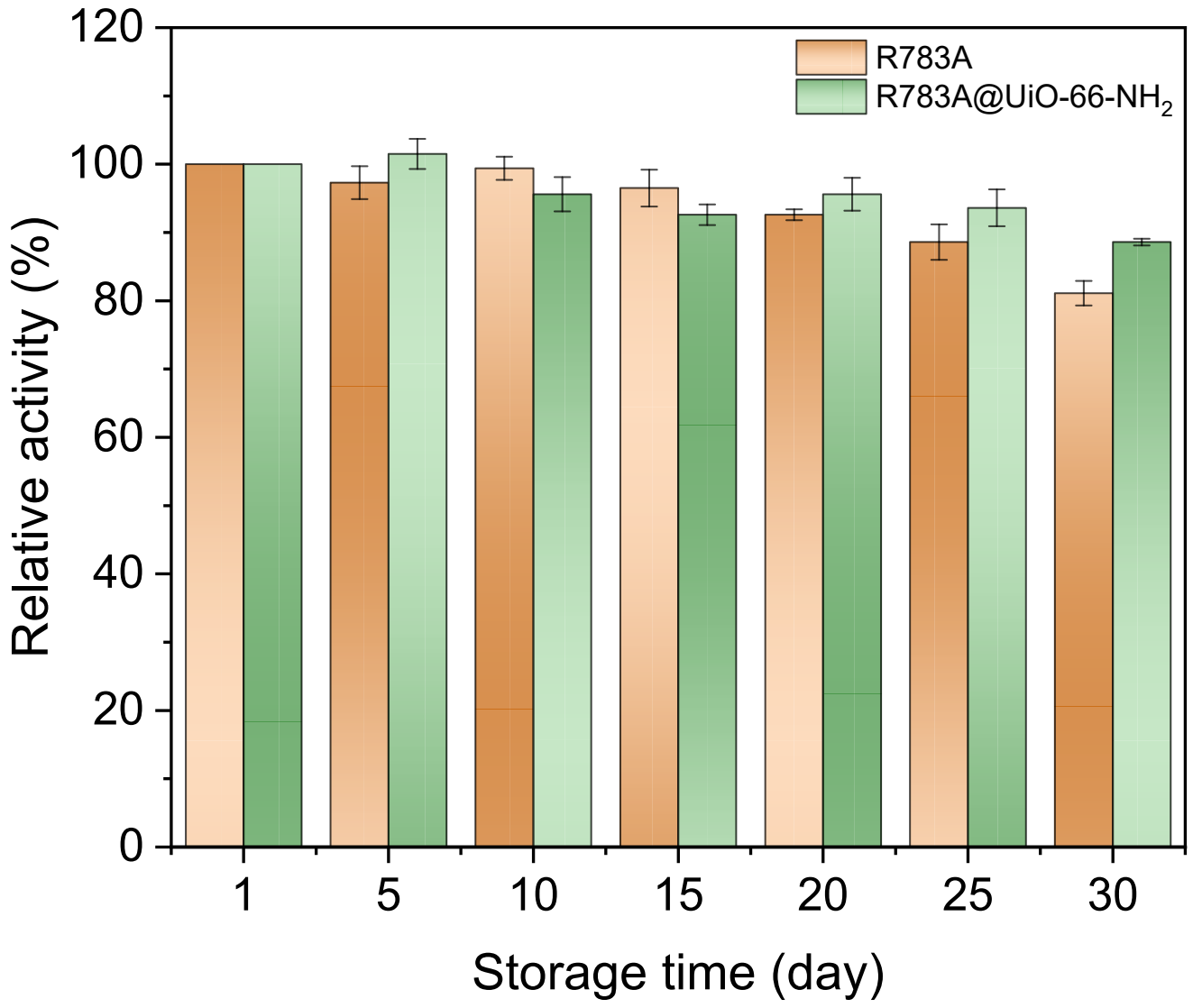

Fig. S2. Storage stability of the free R783A and R783A@UiO-66-NH_2 at 4 °C. Error bars represent means ± SD (n = 3).

Table S1. Adsorption of Cu^{2+} and Zn^{2+} by R783A@UiO-66-NH_2.

Metal ion	Initial concentration (mM)	Concentration after incubation (mM)
Cu^{2+}	1.02 ± 0.02	0.82 ± 0.03
Zn^{2+}	0.99 ± 0.03	0.85 ± 0.02

Data are presented as mean ± SD from three independent experiments.

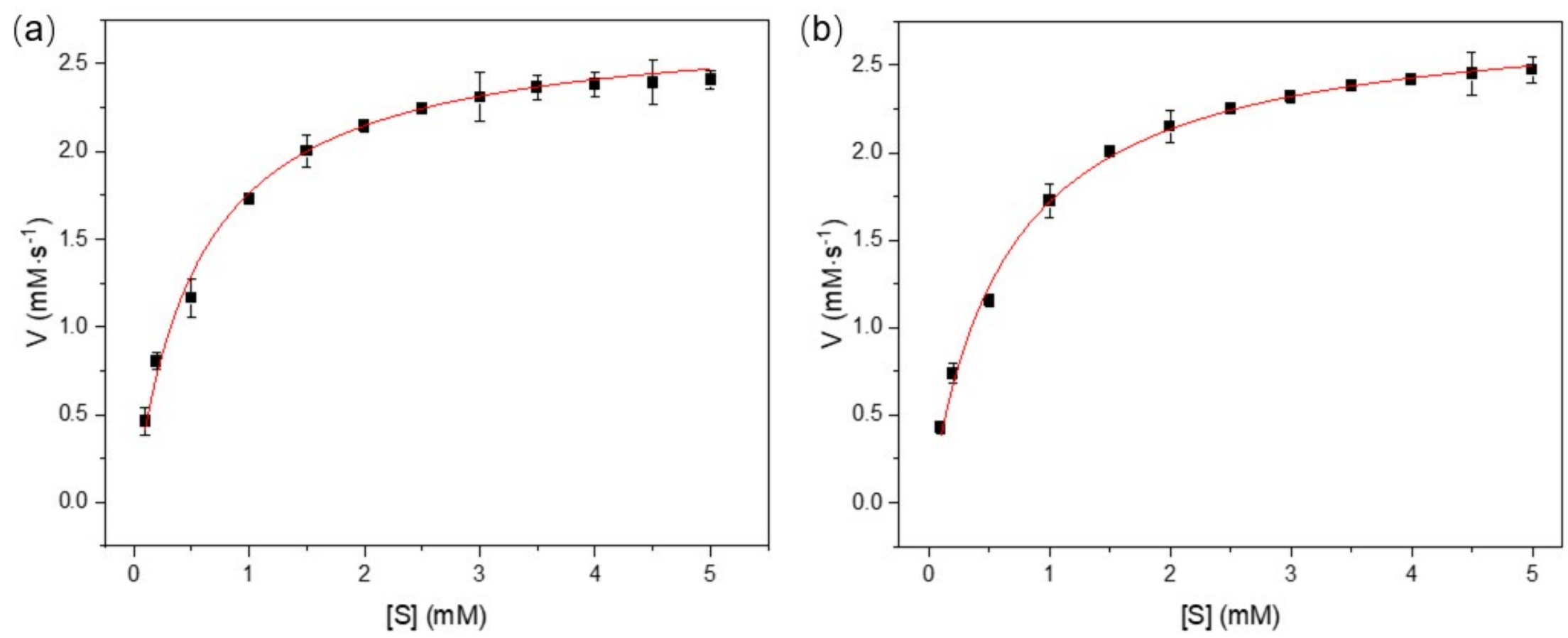

Fig. S3. Michaelis-Menten plots of the free R783A (a) and R783A@UiO-66-NH_2 (b). Error bars represent means ± SD (n = 3).

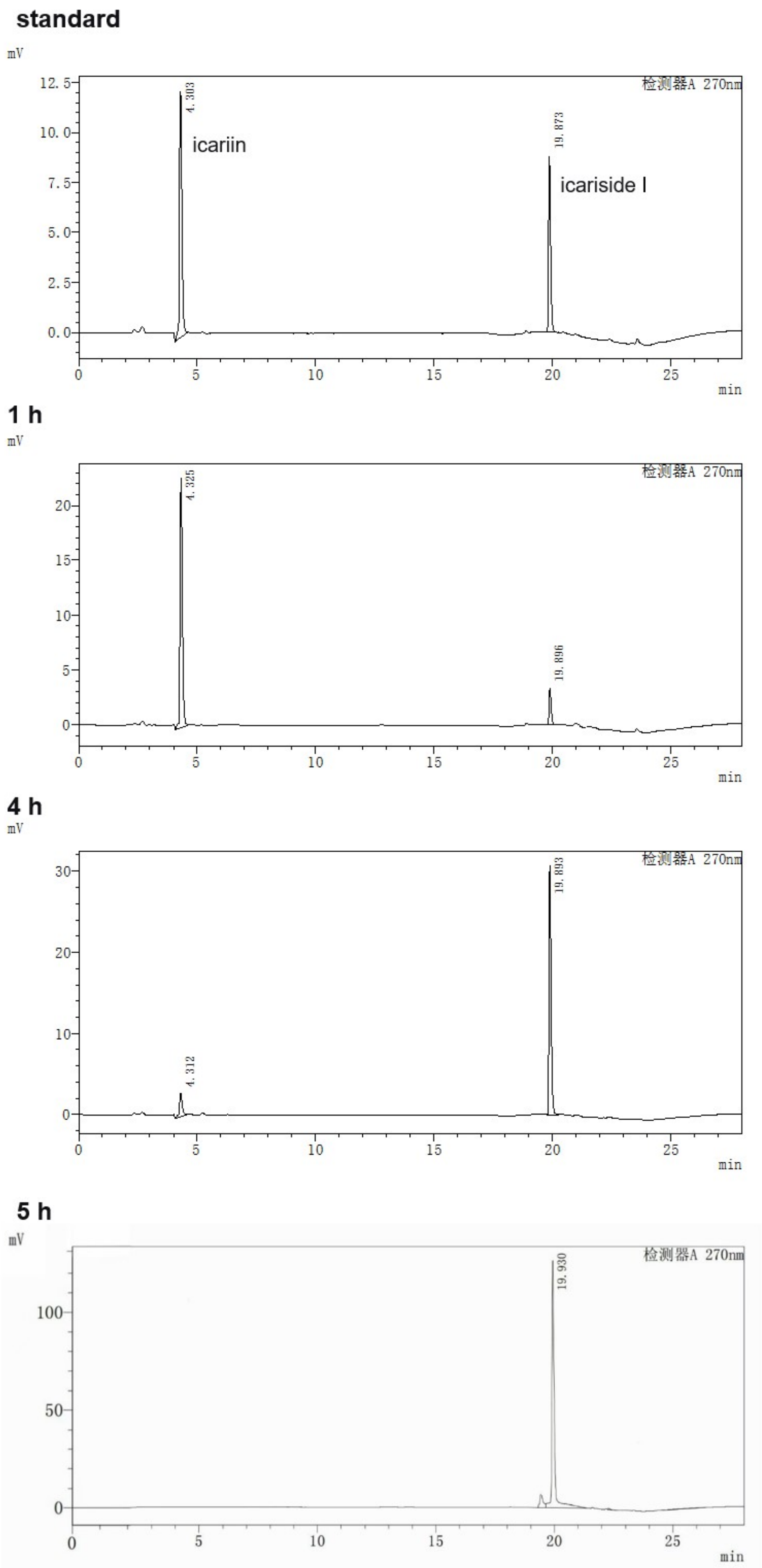

Fig. S4. HPLC spectrogram for analysis of icariside I from icariin.

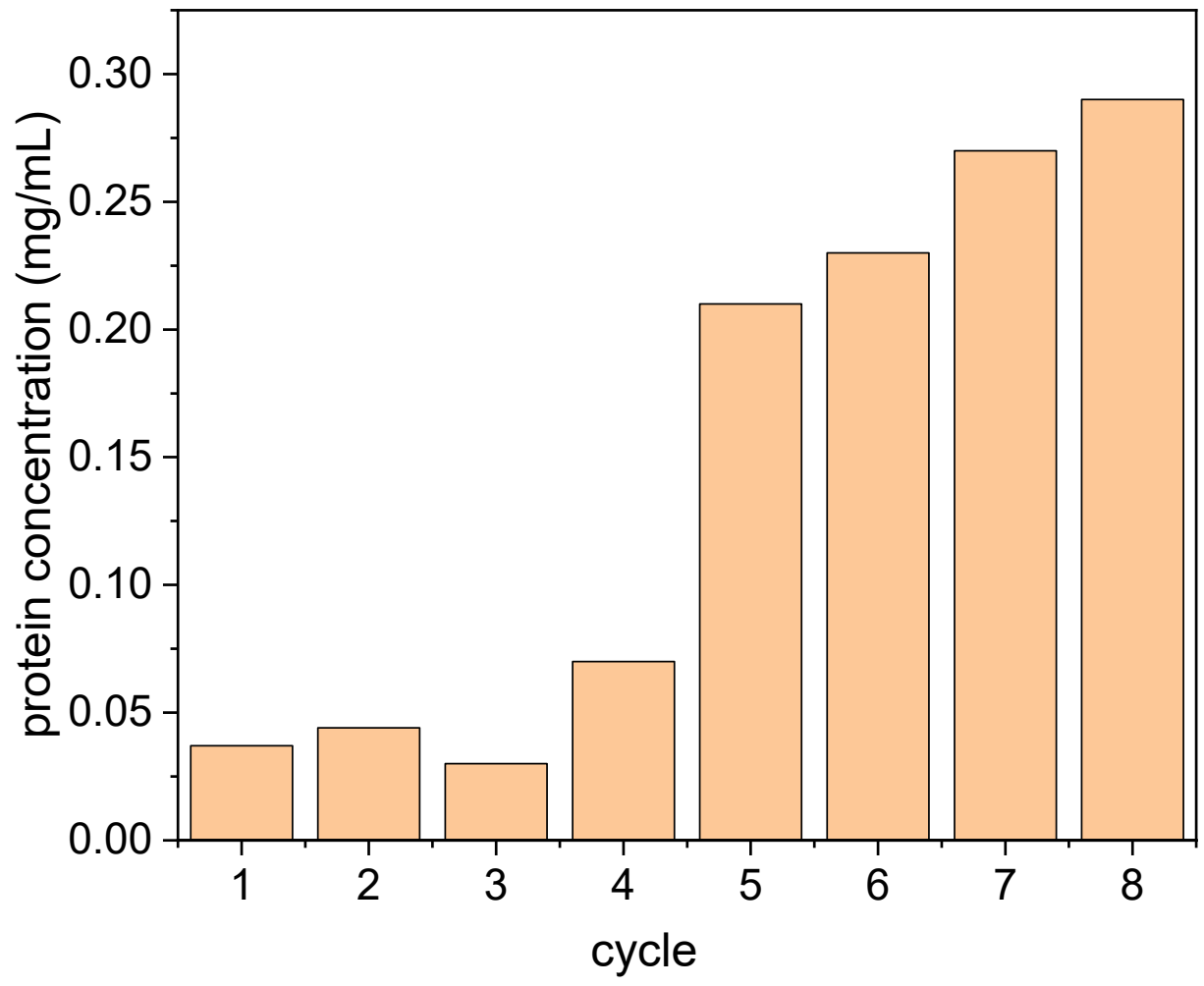

Fig. S5 Concentration of leached protein in the reaction supernatant over 8 cycles.

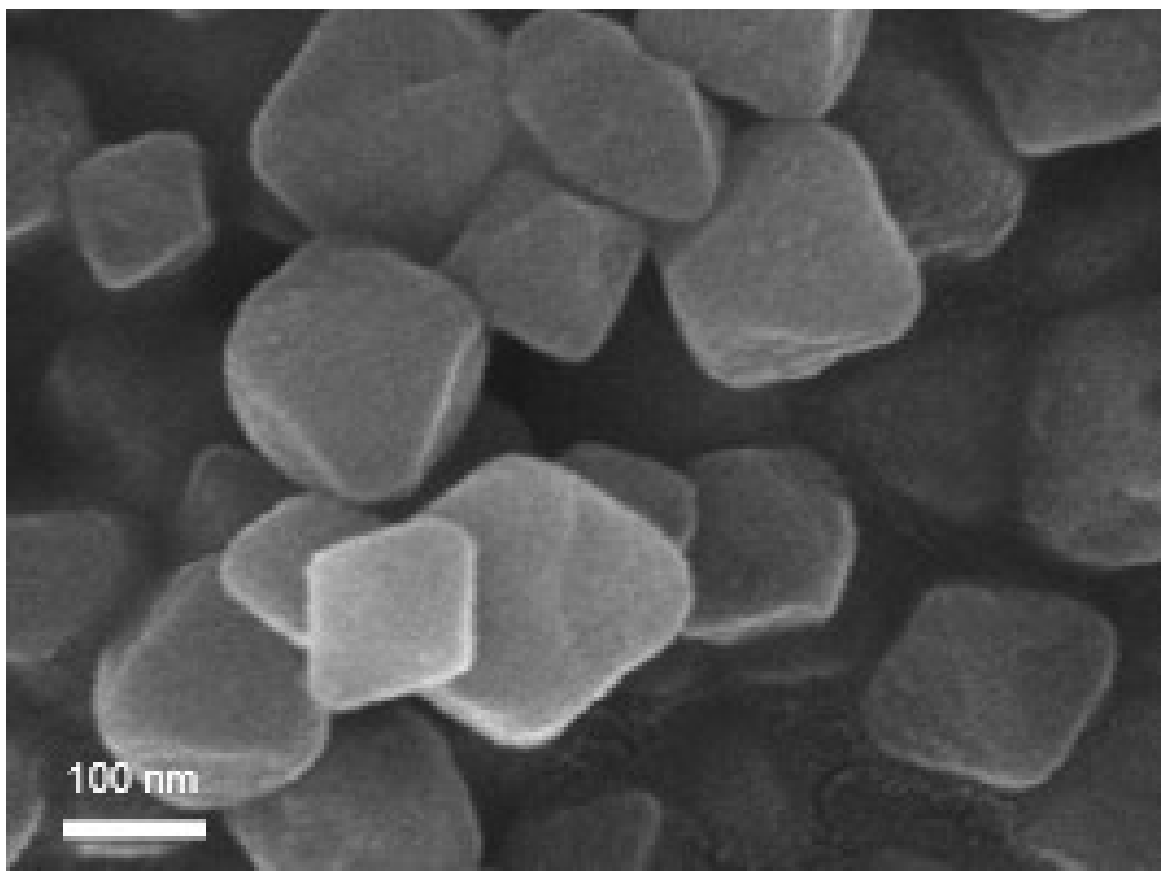

Fig. S6 SEM images of the R783A@UiO-66-NH_2 after the 8th catalytic cycle.

Table S2. Leaching of metal ions from R783A@UiO-66-NH_2 measured by ICP-OES.

Element	Sample mass (g)	Volume (mL)	Concentration in solution (mg L^{-1})	Content in sample (mg kg^{-1})	Content in sample (wt%)
Zr	1	1	0.223	0.223	< 0.0001

Data were obtained from the reaction solution after the 8th cycle of R783A@UiO-66-NH_2 reuse.

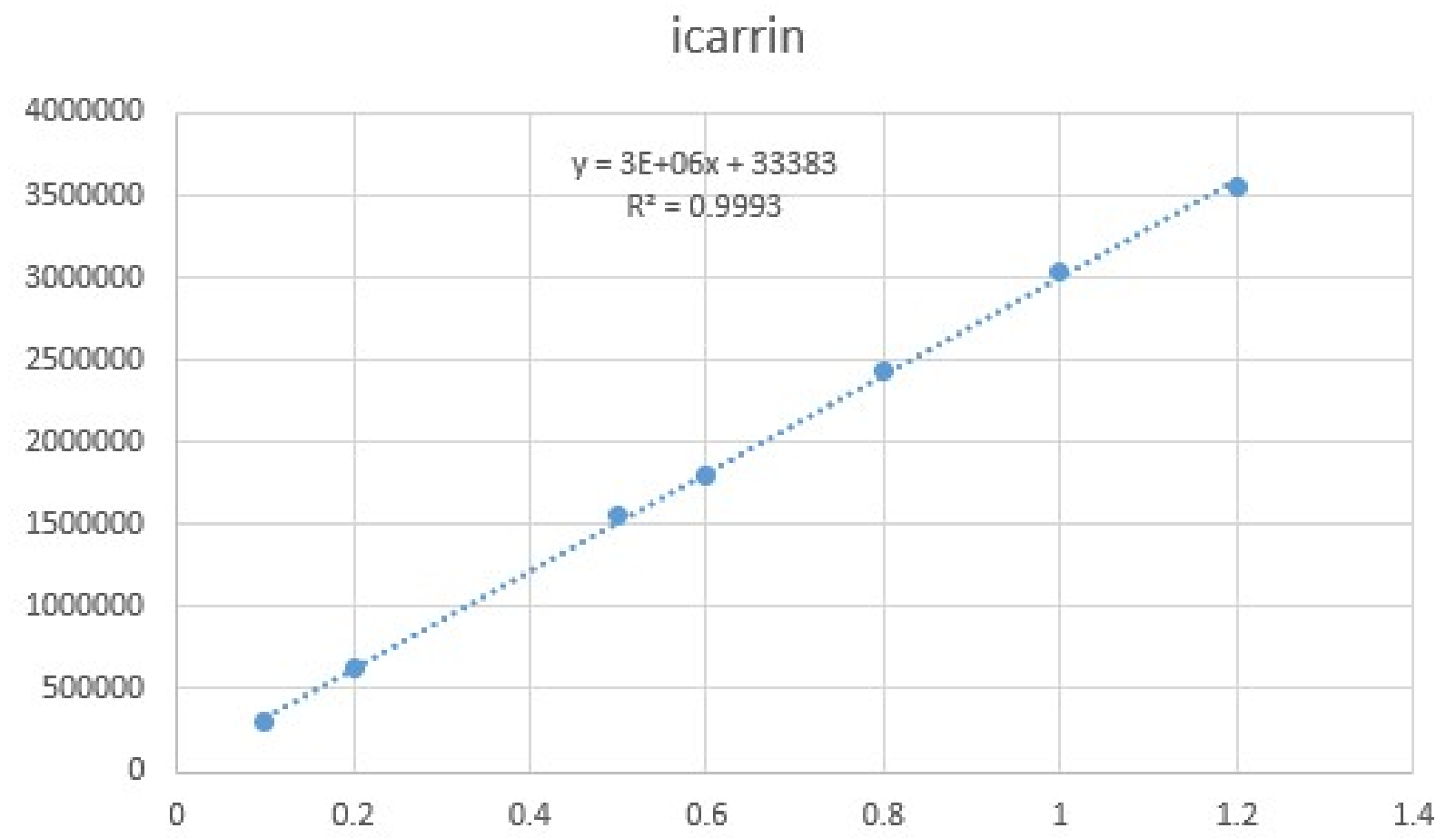

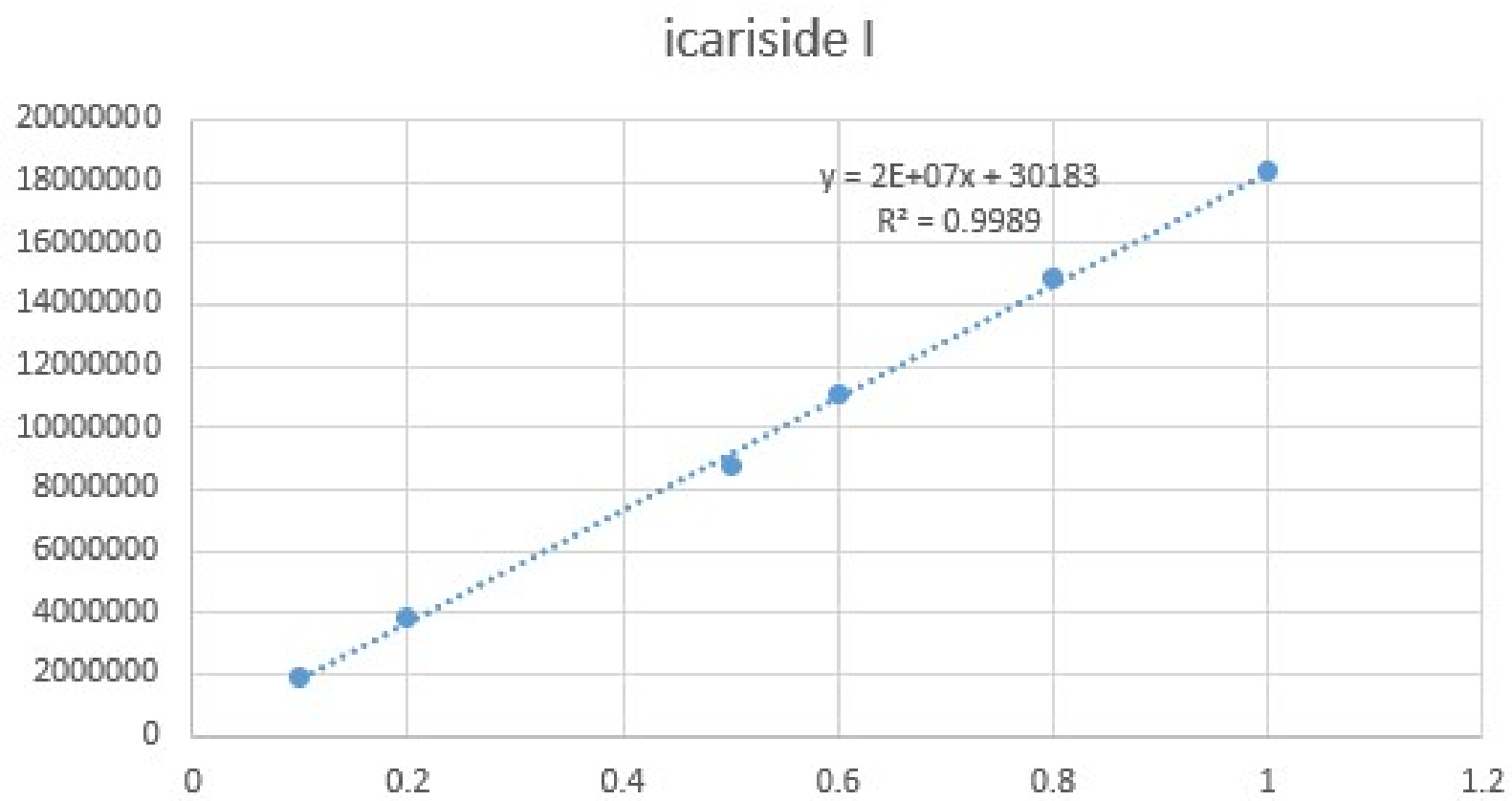

Fig. S7 The calibration curves of icarrin and icariside I.

Table S3. R783A mutant protein sequence.

MKSSNIYSPFDLKCEFTTNPLGVDKKNPIFSWKLRHLEKNEKQTAYQVIVSSSLETINDNIG<br>DVWDTGKVLSSEQVIKYEGKELEPCKVYFWKVRWWDSKDQESPFSVVNTFETGLMNEE<br>NWKAKWITKKEHKYEVYSPDGAPFGLNYTIAYAPMFRKSFSISKKIKRARVYIAGLGLYE<br>LYINGERIGDRVLDPGQTDYKKRVLYTVYDVSKNIRDGKNAIGVILGNGRYVKEYGYDF<br>PKLIIQVLVEYEDDSIEWIVSDESWKTTYGPITLNSLYHGEIYDGRKEIKGWNLPDFDDST<br>WENAILAEPPGGKLYSEIYPPIRITKTIKPIKMWSPEPGTYVYDFGQNYTGWIKIKVRTNES<br>GKEIRIRHAELTYEDGTLNYSTNRTALATDVYITKGEGYEEYEPRFTYHGFRYVEILGYPG<br>VPTLEDIEGKVVHTAVESNGEFICSNELINKIHHNIIWGQLSNLMSIPTDCPQRDERMGWM<br>GDAQLSAEEAIFNFDMIGFYRKYLNDIRDAQKENGSLSDVIPPYWSIYPGDPAWSTAYITI<br>AWYLYQYYGDKYVLEEHYEGFKKYVEFLKKLAPDYIVSFYKYGDWCQPGTVRPKDNSG<br>ELTSTFYFYHDVITLSKIAKLLGKEADYKYYSELADKIKSAFNKKFLKEKAYASIPTELSEE<br>NVKALLEKYPEDIKDFLRQQFTILSSLGMFTSQTLNTLPLYLNLVPEDKVQDVLKTLLEDII<br>IRHDYHLDTGIVATRYIFDVLTSYGYDEVAYKIVNQKTYPSFGYMIEEGATTLWEAWEKL<br>TSTGMNSHNHIMFGSVDAWFYRVIAGVRVGEPGWNKIIFEPHPVGDLKYAKARLNTIKG<br>EVEINWQKTENIFSMRISVPVNSEGEVHVPKLFERFVVKEGDNIIYEKKGDLEENEKYIVIR<br>VGSGSYNFYMEKI
